# Following Up on Employee Surveys: A Conceptual Framework and Systematic Review

**DOI:** 10.3389/fpsyg.2021.801073

**Published:** 2021-12-09

**Authors:** Lena-Alyeska Huebner, Hannes Zacher

**Affiliations:** ^1^Wilhelm Wundt Institute of Psychology, Leipzig University, Leipzig, Germany; ^2^Volkswagen AG, Wolfsburg, Germany

**Keywords:** employee survey, organizational survey, follow-up process, action planning, survey feedback

## Abstract

Employee surveys are often used to support organizational development (OD), and particularly the follow-up process after surveys, including action planning, is important. Nevertheless, this process is oftentimes neglected in practice, and research on it is limited as well. In this article, we first define the employee survey follow-up process and differentiate it from other common feedback practices. Second, we develop a comprehensive conceptual framework that integrates the relevant variables of this process. Third, we describe the methods and results of a systematic review that synthesizes the literature on the follow-up process based on the conceptual framework with the purpose of discussing remaining research gaps. Overall, this paper contributes to a better understanding of the organizational and human factors that affect this process. This is useful for practitioners, as it provides guidance for the successful implementation of this human resource practice. For example, research suggests that it is important to enable managers as change agents and to provide them with sufficient resources.

## Introduction

Employee surveys are widely used in organizations today, and their popularity continues to grow (Church and Waclawski, 2017). Their implementation varies from annual surveys to surveying in shorter intermittent time intervals (e.g., “pulse surveys;” Welbourne, 2016). The purposes of employee surveys include, but are not limited to, enhancing communication between management and staff, giving employees a voice, reducing social distance between management and employees, and intervention/organizational development (OD) (Hartley, 2001; Kraut, 2006). The implementation of an employee survey is not limited to only one of these purposes, but can serve several of them simultaneously (Burke et al., 1996). The success of employee surveys for OD depends heavily on the implementation of a proper follow-up process, that is, the use of the collected data for the initiation of organizational changes (Falletta and Combs, 2002).

Despite its importance, the employee survey follow-up process is often neglected, limiting the effectiveness of this widely used management tool (De Waal, 2014). Many times, organizations view the employee survey process as completed once the data have been collected, consequently failing to properly follow-up on the results and use them as a tool to drive change (Church et al., 2012). Similarly, the literature on the employee survey follow-up process is scarce, as this stage receives less attention by researchers in comparison to numerous studies examining the actual surveying process (Fraser et al., 2009). For example, research has investigated why surveys are conducted at all and what types of items they include (Sugheir et al., 2011), as well as the issue of social desirability in survey responses (Keiser and Payne, 2019). In addition, the sparse literature on the employee survey follow-up process is conceptually fragmented, published across various academic disciplines, and uses inconsistent labels (e.g., employee survey follow-up, feedback intervention). This is especially disadvantageous for practitioners, as it makes it difficult for them to locate reliable evidence-based research, even though employee surveys are a common OD technique (Falletta and Combs, 2002). Also, practitioners lack an extensive overview of relevant factors to consider during implementation, as no comprehensive theoretical model of the process exists. Lastly, there have been reviews on survey feedback interventions or that included such as one of other OD practices, but the most recent work was published over 30 years ago (see Neuman et al., 1989). However, more research on the topic has been conducted since then, but we lack guidance on what variables and domains in this line of research to examine with future studies. Hence, the lack of an updated review of the employee survey follow-up process literature prevents systematic theoretical and empirical research on this important topic and practical progress in this area.

To advance this area of research and practice, we conducted a systematic literature review (Daniels, 2018; Siddaway et al., 2019) on the employee survey follow-up process. First, we define employee surveys, conceptually integrate them into the existing feedback and change management/OD literature, and differentiate them from other feedback practices, such as 360 degree feedback. Describing the nomological network of employee surveys is important because past literature on the topic has been mainly on “survey feedback interventions,” rather than specifically the employee survey follow-up process. Also, differentiating this process from other feedback practices (e.g., 360 degree feedback) demonstrates the necessity of treating this concept as a distinct human resource practice even though it shows similarities to other feedback processes. Second, we developed a conceptual framework to depict the relationships between the relevant variables for the employee survey follow-up process as a change tool. Third, we systematically reviewed and evaluated the literature on the follow-up of employee surveys based on the components of the comprehensive conceptual model. With this approach, the present systematic review explores the following research question: Which variables of our conceptual model have been sufficiently informed by past research and which variables require future research? Finally, we discuss the implications of our review for future research and offer several recommendations for organizational practice.

Overall, our conceptual framework and systematic review contribute to the organizational change and development literature and to practice in four important ways. First, based on a conceptual integration and framework, our review highlights which variables research in this area has investigated, and which variables have been neglected and require further attention. Second, the employee survey follow-up process can generally be categorized as a survey feedback intervention, but is nevertheless a distinct process that deserves focused attention. For example, in contrast to reviews on survey feedback interventions, this review excludes studies conducted with student samples (e.g., Brown, 1972), and on the other hand includes other empirical research conducted on the topic, as for example cross-sectional work (e.g., Church et al., 1995) or qualitative interviews with survey practitioners (e.g., Gable et al., 2010). Third, past reviews on survey feedback are outdated, as more research has been conducted on the topic since then. Hence, our review includes all relevant literature that has been published until today. Fourth, the results of our review are useful for practitioners as they provide an integrated overview of the current state of knowledge on the employee survey follow-up process and of the factors that should be taken into account for the successful implementation of this human resource practice.

## Theoretical Background

**FIGURE 1:**
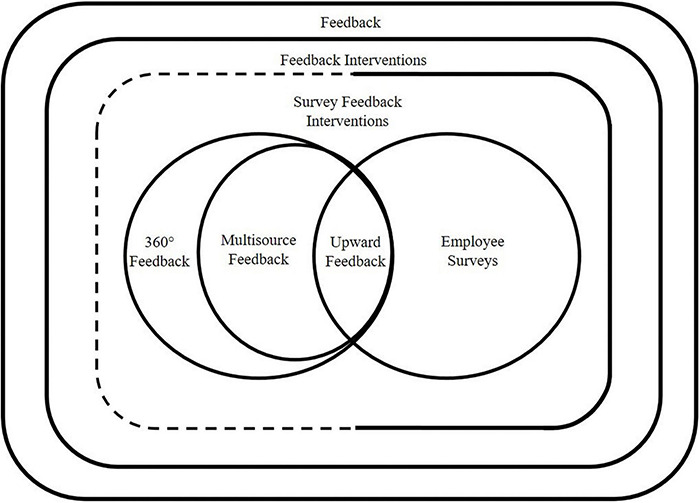
The nomological network of employee surveys. 360 degree-, multisource-, and upward feedback practices are by definition also survey feedback interventions, but generally not explicitly labeled as such in the literature, hence the dotted line.

We begin by conceptually integrating the employee survey follow-up process into the literature on related and overarching topics, including feedback, feedback interventions, survey feedback interventions, and other formats (see Figure 1).

### Feedback

In the broadest sense, an employee survey is a form of feedback, defined as a communication process in which a sender sends a message to a recipient, with the message containing information about the recipient (Ilgen et al., 1979). The term feedback is poorly defined and used inconsistently in the literature (Besieux, 2017). It has been conceptualized and labeled in many different ways, for example as process feedback (how) and performance feedback (what) (Besieux, 2017), as feedback to the individual or the group (Nadler, 1979), or as cognitive (how and why) and outcome feedback (what) (Jacoby et al., 1984). This has led to a plethora of literature on feedback, for example on how to give effective feedback (e.g., Aguinis et al., 2012) or on recipients’ reactions to feedback (e.g., Fedor et al., 1989).

### Feedback Interventions

When feedback is used as an intentional intervention by providing information about a recipient’s task performance and actions being taken by an agent to intervene, this is called a feedback intervention (Kluger and DeNisi, 1996). A meta-analysis on feedback interventions by Kluger and DeNisi (1996) showed large variability in its effects, but there was also large variability in the types of feedback interventions included in the analyses, for example feedback for memory tasks, test performances, and physical tasks.

Feedback interventions have also been considered in the change literature. Guzzo et al. (1985) examined 11 different types of organizational interventions, with feedback interventions being one of them. They found positive effects for this type of intervention practice, yet their scope was broad, too, in that they also included performance appraisal techniques and access to performance data. Nadler’s review (1979) of experimental research on feedback regarding task group behavior, on the other hand, found conflicting results for the effectiveness of feedback interventions to groups. However, feedback was again considered in a broad sense, including feedback for coding or sorting tasks, problem solving exercises, or group discussions.

### Survey Feedback Interventions

When feedback is solicited through the medium of surveying and transferred back to relevant stakeholders for the purpose of diagnosis and intervention, it is called survey feedback (intervention) (Nadler, 1976). Throughout the industrial and organizational (IO) psychology literature, this is generally referred to as “survey feedback,” whereas such interventions can also be applied in different contexts, as for example education or research (e.g., Gehlbach et al., 2018). In the work context, survey feedback interventions entail systematic data collection and feeding the results back to organizational members (Nadler, 1976).

Studies on survey feedback interventions are scattered across the OD literature. Several reviews and meta-analyses have included them as one of many OD interventions. For example, Friedlander and Brown (1974) conducted a review on several different approaches to OD, with survey feedback being one of them. They summarized ten survey feedback intervention studies and concluded that such can have positive effects on the attitudes of those involved. Shortly after, Margulies et al. (1977) summarized six studies relevant to this type of OD intervention and concluded that more research was needed on this technique to understand under which circumstances it produces the most benefits. A few years later, Porras and Berg (1978) and Porras (1979) reviewed four survey feedback intervention studies as one of several different OD techniques, but could not find superiority of this technique over others. Another example of survey feedback relevant for the OD literature is a meta-analysis by Neuman et al. (1989). The authors identified six survey feedback intervention studies out of 84 studies implementing other human processes approaches to OD, meaning such techniques attempt to achieve improved organizational performance *via* improved human functioning. Indeed, the human approach techniques were found to be more effective than techno-structural interventions (i.e., modifications to work or the work environment) in changing organizational attitudes. Lastly, Hopkins (1982) reviewed the use of survey feedback in educational settings and concluded that it is generally useful as a tool in educational organizations. In summary, there is much research on survey feedback interventions, but previous reviews and meta-analyses on this topic have shown mixed results. The majority of authors concluded that more research is needed on this topic, and this assumption holds up until today.

### Other Types of Feedback Practices

Other related human resource practices, for example performance appraisals, such as 360 degree-, multisource-, and upward feedback also rely on systematic data collection and feeding it back to organizational members (DeNisi and Kluger, 2000). Due to the necessity of collecting anonymous feedback, the data for these practices are usually collected with surveys (Bracken et al., 2001), similarly to employee surveys. Therefore, by definition, these practices are survey feedback interventions, but are usually not labeled as such throughout the literature (see dotted line in Figure 1). Also, as the following discussion will show, the specific processes of these practices differ from those of employee surveys.

#### 360 Degree Feedback

One popular practice of performance management is 360 degree feedback, which is a type of performance appraisal that solicits feedback from several sources, mostly for employees in management positions (Atwater et al., 2007). As the name implies, the vertical and horizontal feedback that is collected from multiple rating sources can be conceptualized as a circle. A full circle of feedback constitutes feedback from superiors and subordinates (vertical feedback), peers (horizontal feedback), and self-ratings (Foster and Law, 2006). The goal is to provide feedback to a single person regarding their management qualities (Vukotich, 2014). The two general frameworks in which 360 degree feedback programs are implemented are either for developmental purposes of the rated manager or for administrative purposes, such as promotions (Hannum, 2007).

Generally though, only a small group of people provides feedback. Usually, these are individuals capable of making statements about leadership behaviors because they have worked closely with the rated person. However, the effectiveness of the process is rather limited when the recipients of feedback are left with acting on it without training, which is why it is recommended to have trained facilitators or consultants deliver the anonymous feedback and support managers in understanding the data (Nowack and Mashihi, 2012; Vukotich, 2014).

#### Multisource Feedback

The term multisource feedback (MSF) is often used interchangeably with 360 degree feedback, even though this is not accurate (Foster and Law, 2006). MSF constitutes more than one source of feedback (e.g., self-ratings and peer-ratings), but it must not necessarily involve the full circle of 360 degree feedback. Hence, 360 degree feedback is a type of MSF, but MSF is not necessarily 360 degree feedback (Foster and Law, 2006). However, MSF programs share similar processes with 360 degree feedback initiatives and generally also provide feedback to a single recipient, most often a leader (Atwater et al., 2007). They can also be implemented for developmental or administrative purposes, for example as part of performance appraisal processes (Timmreck and Bracken, 1997).

#### Upward Feedback

Upward feedback is a more narrow form of 360 degree feedback and MSF. It is the vertical feedback derived from subordinates with the purpose of appraising a manager’s performance (van Dierendonck et al., 2007). Upward feedback programs typically include self-ratings of leader behaviors that can then be compared to subordinates’ ratings to help feedback recipients identify development needs and subsequently improve their leadership skills. Similar to 360 degree feedback or MSF programs, upward feedback programs aim to support leadership development or administrative decision-making and entail comparable processes (Atwater et al., 2000).

#### Comparing Other Feedback Practices to Employee Surveys

Employee surveys are similar to the above mentioned human resource feedback practices, but are nevertheless distinct in their processes and goals. Their most overlap occurs when an employee survey contains items on leadership behavior, specifically direct leaders. In such a case, the employee survey functions as upward feedback to managers in addition to the assessment of general work conditions (Church and Oliver, 2006). The most prominent differences between the various human resource feedback practices and the employee survey is the type of feedback that is solicited and the handling of the data following the survey. Employee surveys only utilize vertical feedback, meaning feedback is carried up the organizational hierarchy starting at the bottom. They entail formal feedback derived from large groups of or all employees in an organization (best case at least from a representative sample), and the results are aimed at evaluating general work conditions. The goal is therefore not to evaluate a specific employee’s leadership skills, but to obtain feedback from a wide range of employees on more general work-related topics (Bungard et al., 2007).

The employee survey follow-up process then entails using the group-level feedback data for organizational change initiatives. Some organizations choose to implement top–down initiatives in reaction to survey results in which management or other stakeholders review the data at a higher and aggregated level than that of single teams. They then decide on overarching action plans for the whole company or certain departments, such as the implementation of new performance appraisal systems, overhauling internal communication, or changing the company strategy (Linke, 2018). Such top–down approaches are not the focus of this review, but the interested reader is referred to different case study descriptions (see e.g., Chesler and Flanders, 1967; Goldberg and Gordon, 1978; Rollins, 1994; Falletta and Combs, 2002; Feather, 2008; Tomlinson, 2010; Costello et al., 2011; Cattermole et al., 2013).

The focus of this review is the bottom–up approach to change, which focuses on employee involvement and participation and is of a more decentralized nature (Conway and Monks, 2011). The employee follow-up in line with this approach entails the discussion of psychosocial working-environment data between managers and their teams and having a dialogue about results that pose areas with need for action. Ideally, action planning and proper action plan implementation should follow these discussions (Welbourne, 2016).

As mentioned previously, such follow-up steps after the survey are oftentimes neglected in practice (Church et al., 2012). One reason for this could be that employee surveys generally have different purposes in comparison to 360 degree, multisource, and upward feedback approaches. They are mostly used for OD or assessment purposes (Hartley, 2001). They are much less likely to be tied to personal rewards, such as promotions of specific managers. Hence, the responsibility to review the data and to implement changes based on it does not lie as clearly with managers as it does with the feedback practices described above.

Overall, there is little empirical evidence regarding the follow-up on employee surveys, and the research that is available is scattered and labeled inconsistently (e.g., employee satisfaction survey, opinion survey, engagement survey). As noted above, researchers have offered reviews and meta-analyses on different types of feedback, feedback interventions, and specifically survey feedback interventions. From a holistic perspective, however, the results of these reviews are mixed and inconsistent, calling for a systematic review on the distinct concept of the employee survey follow-up. In the following section, we offer a conceptual framework for presenting research on this topic.

## A Conceptual Framework of the Employee Survey Follow-Up Process

**FIGURE 2:**
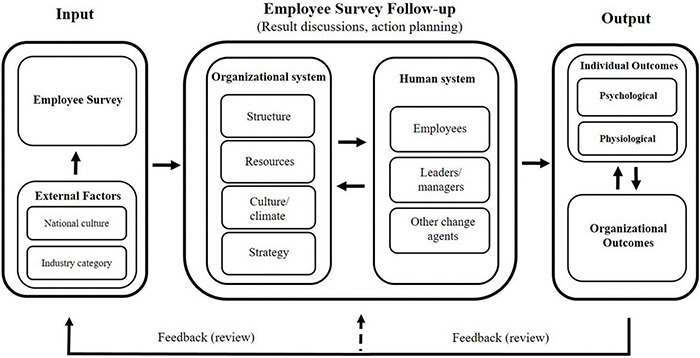
Conceptual framework of the employee survey process, specifically the follow-up process. Variables listed as external factors serve as examples; list is not exhaustive.

We developed a conceptual framework of the employee survey process, with particular focus on the follow-up (see Figure 2). For its development, we drew from existing theory and research. Mainly, the OD/change and organizational behavior literature informed this model, more specifically models proposed by Nadler and Tushman (1980); Burke and Litwin (1992), and Porras and Robertson (1992).

Nadler and Tushman’s Congruence Model of Organizational Behavior (1980) informed the general structure of our model with its input-, transformation process-, and output approach to behavioral systems in an organization, which is in alignment with open systems theory (Katz and Kahn, 1978). According to their conceptualization, there are inputs for the behavioral system (i.e., the organization). This behavioral system consists of specific organizational elements and produces behaviors that ultimately lead to certain levels of organizational performance (i.e., outputs).

This systems and transformation view of the organization is applicable to the employee survey (follow-up), as this process itself is an approach to identifying and solving organizational problems. Specifically, the post-survey follow-up is an organizational transformation process fed with data from certain input sources, such as the employee survey (Falletta and Combs, 2002). This transformation process emerges, like any other organizational process, through the interaction of human and organizational factors and the resulting behaviors (Nadler and Tushman, 1980). Lastly, such systems put forth outputs that can be categorized into organizational and individual performance (Nadler, 1981).

Two other common and popular change models inform the more specific variables of the model; Burke and Litwin’s Model of Organizational Performance and Change (1992) and Porras and Robertson’s Change-Based Organizational Framework (1992). Figure 2 attempts to portray the primary variables and components relevant to the employee survey follow-up process. Below we will describe each component of the model in more detail.

### Input

#### The Employee Survey

The employee survey itself produces the necessary data for all subsequent steps (i.e., teams receive their results and plan actions based on them) (Linke, 2018), hence it can be considered as an antecedent of the survey follow-up process. Much research has been accumulated on survey development and administration, but it stands mostly in isolation from the steps following the actual survey, meaning most studies do not connect this knowledge to the survey follow-up steps, creating a disconnect between these bodies of literature.

#### External Factors

Besides the survey delivering data as input for the follow-up process, there are also factors external to the organization that provide input for the follow-up. As other researchers have noted, external factors affect and sometimes initiate organizational change (Burke and Litwin, 1992; Porras and Robertson, 1992). These factors can include any outside conditions that influence the organization, for example political circumstances, culture, marketplaces, or even industry category (Burke and Litwin, 1992). These external factors represent the context in which the employee survey is embedded and therefore also have an effect on the employee survey and follow-up. For example, the culture of the country that the company resides in will most likely influence what kind of questions are asked in an employee survey (e.g., collectivist vs. individualistic cultures). Culture most likely also influences participation rates in an employee survey (e.g., there might be low participation rates when the survey content does not fit the cultural context).

### The Employee Survey Follow-Up Process

Consistent with Porras and Robertson’s (1992) Change-Based Organizational Framework, we identified two main factors that are relevant for the follow-up process: The work setting (i.e., organizational system) and its organizational members (i.e., the human system).

#### Organizational System

There are many ways to think about the components of an organization, hence there is no one way agreed upon description (Nadler and Tushman, 1980). Generally, these components refer to the organizational arrangements that characterize how an organization functions. We have listed the components we deemed most important for the implementation of employee surveys and their follow-up: Structure, resources, culture/climate, and strategy. Structure refers to the arrangement of people and their functions into different levels of responsibility and authority (Duncan, 1979). As employee survey follow-up processes take place in work groups, the structure of an organization becomes defining for the constellations in which the process is carried out (Nadler, 1980). Resources refer to any organizational, physical, psychological, or social aspects of work that help achieve work goals (Demerouti et al., 2001) and are hence also relevant for all work-related processes, such as employee surveys and their follow-up. Culture and climate are related constructs, with culture referring to the collection of rules, values, and principles that guide organizational behavior. Climate refers to the collective impressions, feelings, and expectations of members in a team or work unit (Burke and Litwin, 1992). Culture has long been recognized to play an important role in OD (Beer and Walton, 1987), and with the follow-up process being a team-level task, there is reason to believe that especially the climate in a work unit will affect this process as well. Strategy is how an organization intends to achieve effectiveness over an extended time frame (Burke and Litwin, 1992), and the literature on employee surveys suggests that the goals of employee surveys (including their follow-up) should be aligned with the company’s strategy (Falletta and Combs, 2002). Generally, surveys can and should also be used to support the organization’s strategy (Macey and Fink, 2020).

#### Human System

The human system refers to any participants and change agents involved in the process of the employee survey and its follow-up. Leaders are important change agents in OD (Beer and Walton, 1987), and the employee survey (follow-up) process requires dedication from top management down to direct supervisors (Knapp and Mujtaba, 2010). Whereas the top–down approach to change is of a strategic and centralized nature and managed from higher levels of the organization, the bottom–up approach to change focuses on employee involvement and participation (Conway and Monks, 2011). Hence, employees are also important to the process und take on the role of change agents.

Lastly, whereas some literature on employee surveys recommends that only employees and team leaders are present during the feedback and action planning meetings (see e.g., Knapp and Mujtaba, 2010), some sources recommend that trainers or consultants help facilitate during the process by supporting managers in making sense of the data and engaging in action planning discussions with their teams (see e.g., Bungard et al., 2007; Linke, 2018). Consequently, other change agents besides managers and employees can play an important role in the process.

### Output

Output is what the organization produces, more specifically its performance (Nadler and Tushman, 1980), but there is a lack of consensus as to what constitutes a valid set of performance criteria in an organization (Ostroff, 1992). There is, however, general agreement that performance is multi-dimensional and applies to the multiple levels of an organization (i.e., the individual-, team-, and organizational level) (Sonnentag and Frese, 2002). In the context of this research, we drew from the above mentioned change models by Nadler and Tushman (1980); Burke and Litwin (1992), and Porras and Robertson (1992) and differentiate between individual (psychological vs. physiological) and organizational outcomes, assuming that these two can influence each other.

### Feedback Loops

The feedback loops pertain to the process of reviewing developed action plans and evaluating them regarding their effectiveness and sustainability. This helps create accountability and guide future decisions regarding readjustment of action plans or the necessity to develop additional action plans based on the current survey cycle (see smaller loop circling back to the follow-up process in Figure 2; Bungard et al., 2007).

The second loop connects back to a new survey cycle, restarting the process of action plan development based on newly collected data (see Figure 2). This feedback loop informs the future survey and follow-up process in that new action plans can be informed by the outcomes of previous action plans. For example, if an action plan was not successfully implemented, an additional action might be developed. Also, past research has shown that previous experiences with change initiatives can shape attitudes toward future change initiatives, such as levels of trust in future change programs (Bordia et al., 2011). More specifically, past research suggests that the quality of handling survey data and conducting a follow-up process might influence attitudes toward future surveys, including perceptions of its usefulness (Thompson and Surface, 2009) or the intent to participate in future surveys (Rogelberg et al., 2000).

## Method

### Literature Search

From September 2020 to December 2020 and in June 2021, we conducted several comprehensive literature searches in Google Scholar and PsycInfo. We used the search terms “employee survey,” “survey feedback,” “organizational survey,” “employee engagement survey,” “employee opinion survey,” “employee satisfaction survey,” “survey feedback intervention,” and “survey key driver analysis.” We also searched “upward feedback” as we expected for this term might not only refer to traditional upward feedback programs, but that this term might also put forth research that refers to vertical feedback.

**FIGURE 3:**
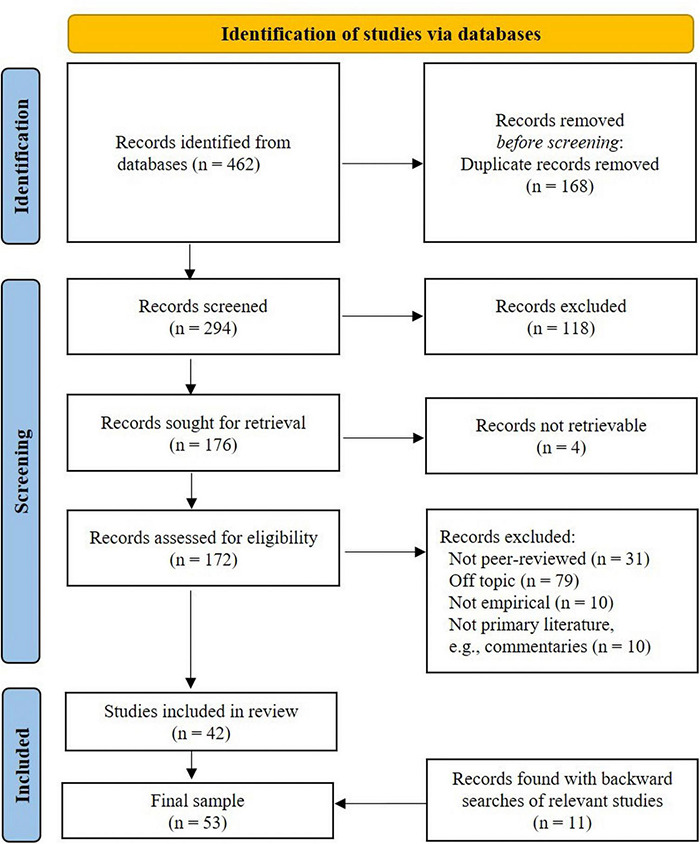
Systematic literature review process.

The literature seldom discusses the follow-up process without the preceding surveying process. Therefore, during the initial phase of the database search, we included all titles that indicated a discussion of employee surveys in general. An important distinction was whether the title of the study indicated merely the use of surveys as the data collection method for other research purposes or whether the record discussed the process of conducting an employee survey. This especially posed a challenge for this review, as surveys are the most popular method of research in psychology (Dillman et al., 2014). The search resulted in 462 initial records (see Figure 3).

### Inclusion and Exclusion Criteria

Following the Preferred Reporting Items for Systematic Reviews and Meta-Analyses (PRISMA-P) protocol (Moher et al., 2015), we screened all articles.^1^ The inclusion criteria applied during the scanning of abstracts and full texts were that the record (1) primarily discusses the bottom–up approach to organizational change in the context of the employee survey follow-up process, which constitutes the group discussion of fed back psychosocial data, (2) constitutes primary empirical literature published in peer-reviewed academic journals or book chapters of edited books, and (3) it is written in English or German. Regarding point (2), we chose to not include gray literature (e.g., dissertations, conference papers) to ensure a sufficient level of quality of the included literature, which is guaranteed by the peer-review process of academic journals and of edited books.

We excluded general books on the matter because, as a common and popular human resource practice, there are numerous books on employee surveys, which are ultimately based on the empirical literature we summarize in this review. The employee survey process at organizations is defined by the dynamics between managers and teams, and this is different to a teacher and student context. Hence, we excluded research conducted in educational settings when it was conducted with teacher and student samples (e.g., Brown, 1972; Hand et al., 1975). We did, however, include studies in educational settings when the survey feedback was used among educational staff (e.g., between principals and teachers) for the development of the educational institution as an organization (e.g., Miles et al., 1969). We also excluded non-primary literature, such as book reviews and commentaries, because these are also based on the primary work we summarize in this review. Finally, we searched the references of relevant papers until no new records were identified, which resulted in an additional 11 records. The final sample constitutes 53 records published between 1952 and 2021.

For each paper, we tabulated and extracted the following information: Author(s), year of publication, the research field the study was published in, the terms used to describe the employee survey/the follow-up process, the study type/analytic methods, and a short summary of findings (see Appendix). We also coded all records according to which components of the conceptual model they inform. When the record contained information pertaining specifically to a variable as listed in the conceptual model, the record was coded and listed accordingly in Table 1. In addition, we coded records according to whether the study indicated the involvement of an external change agent, more specifically the level of involvement of another change agent. We coded a study as indicating low change agent involvement when there was no involvement or little involvement either during the preparation stage of feedback meetings or during the actual feedback meetings. We coded a study as indicating medium involvement of a change agent when such supported managers thoroughly either during the preparation phase (e.g., thoroughly briefed managers on how to conduct meetings) or during the actual feedback meetings (e.g., they moderated the feedback meetings for or with managers), or when they supported moderately during both phases. We coded the record as indicating high involvement of an external change agent when they thoroughly supported managers during preparation and during the actual feedback meetings.

**TABLE 1:** Reviewed empirical studies coded according to which components of the conceptual model of the employee survey follow-up process they inform.

				Number of studies	Citations*
Input		Employee survey		1	17
		External Factors		0	–
Employee survey follow-up	Organizational system	Structure	Family group	48	1; 2; 3; 4; 5; 6; 7; 8; 9; 10; 11; 12; 14; 15; 18; 19; 20; 21; 23; 24; 25; 26; 27; 28; 29; 30; 31; 32; 33; 34; 35; 36; 37; 38; 39; 40; 41; 42; 43; 44; 45; 46; 47; 48; 49; 50; 51; 52
			Peer-/intergroup	5	3; 16; 29; 46; 47
			Other	2	19; 20
			Not applicable	4	13; 17; 22; 53
		Resources		3	18; 22; 53
		Culture/climate		1	9
		Strategy		0	–
	Human system	Employees		5	3; 12; 26; 42; 43
		Leaders/managers		9	2; 3; 8; 15; 18; 31; 42; 43; 49
		Other change agents**	Low involvement	14	5; 6; 7; 12; 14; 15; 24; 28; 30; 37; 38; 39; 42; 43
			Medium involvement	22	1; 2; 4; 8; 10; 16; 18; 19; 20; 23; 27; 34; 35; 36; 44; 45; 46; 47; 48; 49; 51; 52
			High involvement	12	3; 9; 11; 21; 25; 26; 29; 32; 33; 40; 41; 50
			Other information	3	2; 31; 35
			Not applicable	4	13; 17; 22; 53
Output	Individual outcomes	Psychological		38	1; 3; 4; 5; 6; 7; 8; 9; 10; 11; 12; 13; 14; 15; 16; 18; 19; 20; 21; 27; 28; 29; 30; 32; 34; 35; 36; 37; 38; 39; 40; 41; 42; 43; 46; 47; 48; 49
		Physiological		4	5; 19; 20; 21
	Organizational outcomes			9	1; 5; 7; 10; 14; 29; 34; 42; 43

Total number of studies: 53. *For according citations, see Appendix. **Studies were coded according to involvement levels of additional change agents other than managers: Low (no involvement or little involvement before or during feedback meetings); medium (thorough involvement either before or during feedback meetings or moderate involvement during both phases); high (high involvement before and during feedback meetings).

Coder(s) also recoded 10% of the studies to check their consistency (Daniels, 2018).^2^

Six records indicated that data was used for multiple publications (i.e., constituting three unique publications) and were marked as such in the Appendix. We suspected eight additional records to constitute only four unique publications based on the analog study design descriptions. We were able to acquire contact information from at least one author of two (i.e., four) manuscripts. One confirmed the multiple use of data and one was not able to provide information due the long time that had passed since publication.

## Results

In the following, we summarize and integrate the findings derived from the records we identified *via* our literature searches and structure them according to the components of our conceptual model with the purpose of revealing domains in which our evidence-based knowledge remains underdeveloped.

### Input

#### The Employee Survey

None of the studies included in this review investigated the characteristics of the employee survey as antecedents to the follow-up process. A variety of different questionnaires served as the basis for follow-up activities, and there was also much variety in the extent of information that the authors provided about the questionnaires. Whereas some provided many details and item examples, others merely named the survey. In some instances, the questionnaires were matched to the specific context and circumstances of the organization, for example to a military setting [the Army’s General Organizational Questionnaire (GOQ); Adams and Sherwood, 1979], or to mining and milling (the Work Attitudes Survey; Gavin and Krois, 1983).

Overall, the surveys contained items regarding a variety of psychosocial constructs relevant to the workplace. Examples include, but are not limited to, job demands, control at work, social interactions, leadership, and commitment to the organization (Björklund et al., 2007), as well as rewards, communication, quality of management, participation, employee satisfaction, organizational climate, and effectiveness (Amba-Rao, 1989). More examples include items on response to stress, the need for work development, and perceived work environment (Elo et al., 1998), as well as items regarding quality of work life, individual morale, individual distress, supportive leadership, role clarity (Jury et al., 2009a,b).

Results by Gavin and McPhail (1978) of an implemented employee survey at an Admissions and Records Department suggest that it might be more beneficial to use items developed for the specific context of an organization, rather than general organizational climate measures, as those items improved more in comparison to the general items. Consequently, the authors suggested that tailored survey interventions might be more effective than global initiatives. Similarly, Adams and Sherwood (1979) also suggested that items tailored to the specific context might be more beneficial than general items.

Lastly, one study discussed the usefulness of survey key driver analysis (SKDA) for managers in the survey data analysis process, which is a statistical procedure to identify topics that can be prioritized for action planning among a variety of other measured topics in a survey. More specifically, the identified key drivers are most highly associated with the outcome (oftentimes employee engagement). Cucina et al. (2017) called for the moratorium of this practice, which evoked a series of commentaries (see Hyland et al., 2017; Johnson, 2017; Klein et al., 2017; Macey and Daum, 2017; Rotolo et al., 2017; Scherbaum et al., 2017). Similarly, some authors have suggested that managers do not need statistical training to recognize significant differences, but instead can deal best with their data by examining percentages of favorable and unfavorable results and comparing them to other departments or past survey results (Dodd and Pesci, 1977). However, in some studies, managers received survey results prepared through survey key driver analysis (SKDA) (e.g., Griffin et al., 2000; Ward, 2008).

In summary, whereas all studies provide a mostly sufficient description of the employee survey that was used for the intervention, we recognized a disconnect between the survey items and their significance as antecedents to the action planning process. It is reasonable to assume though that the questionnaires help participants structure their subjective feelings and guide subsequent action planning by providing relevant concepts for discussion. Also, the way the data is prepared by or for managers most likely also affects the subsequent action planning process.

#### External Factors

None of the studies included in this review explicitly examined external factors, but as we described in earlier sections, such are complex and difficult to define and measure. One important factor to consider could be, for example, the national culture in which the organization is embedded. None of the empirical studies examined the employee survey follow-up process from a cross-national perspective, but our review yielded studies conducted in Australia, Germany, Finland, South Africa, Sweden, the United Kingdom, and the United States. Also, the studies included in this review were implemented in a variety of different industries, as for example military, banking, schools, hospitals, manufacturing, and mining, but none of them examined effects across different industries. Therefore, our results suggest that the role of external factors is yet to be explored in the context of employee surveys and their follow-up.

### The Employee Survey Follow-Up Process

#### Organizational System

##### Structure

The classic structure for the implementation of employee surveys is the waterfall design in work families. Within this approach, higher level feedback sessions serve as role models for lower level work groups, and results are presented to and in the according work families (i.e., a manager with her/his subordinates) (Nadler, 1980). Most reviewed intervention studies made use of this model (see Table 1); Adams and Sherwood (1979) for example reported some improvements following an employee survey conducted in a military setting with strong hierarchical structures, which matched the classic waterfall and work family design.

However, some researchers have suggested the superiority of other structure models for survey feedback meetings. For example, Alderfer and Ferriss (1972) found that higher level managers denied their problems in feedback meetings, while exhibiting a decline in workplace morale. The authors suggested that the traditional work family model might not be the most effective way to conduct survey feedback meetings, as it might lack psychological safety for participants. Instead, it could be useful to first conduct peer meetings, which can be followed by work family meetings. One year later, Alderfer and Holbrook (1973) followed up with a study in which they implemented a peer-/intergroup model instead of a work family design with which they found some positive effects: Individuals that shared a common organizational fate, for instance because they had similar tasks, but they did not have direct authority relationships, were brought together for the employee survey follow-up meetings. Managers also met among each other, and these meetings were followed-up by intergroup sessions in which members of the different systems at different levels of authority interacted. The authors proposed that there might be less hesitance of employees to speak up in such meetings because direct managers are not present.

Eklöf et al. (2004) compared other types of structure models during which feedback was provided by a trained ergonomist: Individually to each person in the group, to only the group supervisors, and to the entire group with the supervisor present. Results suggested potential benefits in giving feedback to only supervisors. This was the most cost effective intervention group, as it resulted in a higher average number of psychosocial modification types per individual (i.e., different types of modifications to the workplace) and required the least time investment. It is important to note though that the average number of psychosocial modification types per individual decreased for all groups during this intervention, but the supervisor feedback group merely showed the least decrease.

In summary, research suggests that other implementation structures besides the classic waterfall and work family design for the employee survey follow-up could be useful, but we require additional research to compare and further explore such implementation strategies.

##### Resources

Only three of the studies specifically examined resources in the employee survey follow-up context. Dodd and Pesci (1977) found that managers who received feedback training seemed to conduct more feasible, measureable, visible, and timely action programs than managers without training. Trained managers were also more likely to improve employee attitudes and morale through the feedback intervention. Wiley (2012) surveyed 31 survey practitioners from a sample of large organizations, and the top three barriers to effective post-survey action planning named were execution (following through), importance (lacking attention by executive management), and resources (especially time, but also lacking training, technical, and financial resources). Lastly, Fraser et al. (2009) interviewed 18 managers from large multi-site companies that had implemented employee surveys in the past. Results indicated that important resources for the implementation of a successful follow-up process included a clear action purpose of the survey itself, senior management endorsement of the implementation, experienced leaders, and the support of trained change agents to drive the process.

Some other studies mentioned almost in passing different types of resources (e.g., time, financial resources, training) that affected the employee survey process. For example, participation in the survey intervention implemented by Elo et al. (1998) was voluntary, but sessions were held at times where shift workers could participate immediately before or after their working hours, and the company paid compensation to those who participated. Church et al. (2012) provided some qualitative data from employees who reported that action was not taken based on their survey results, and they named a lack of commitment by managers to follow through as one of the reasons. The participants also named the lack of other resources, including time, funding, or manpower. Lastly, Baker et al. (2003) reported in their study that some managers noted that the pressures of their daily work made it difficult to disseminate the results to the entire staff.

Overall, resources seemed to not find much attention in the reviewed literature. The reason for this neglect could be that the majority of study contexts might not have suffered from lacking resources because organizations consenting to collaborate in research and the research teams implementing the intervention are likely to ensure that the research can be carried out appropriately with sufficient resources.

##### Culture/climate

Similarly to resources, organizational culture/climate was given little attention throughout the literature. An exception was a study by Bowers (1973), in which he examined organizational climate as a mediator. He found that the positive effects of survey feedback on measures of organizational functioning were weaker when controlling for climate. Other anecdotal descriptions provide inferential information about the importance of culture/climate to the employee survey follow-up. Swanson and Zuber (1996) described the hostile organizational culture of the mailing company that their intervention was attempted to be implemented in. There was high turnover with managers routinely being fired or demoted without clearly stated reasons, which resulted in managers maintaining low profiles and not speaking up. Top management generally showed defensiveness toward the survey reports and an unwillingness to change. Overall, the organizational culture was hostile, hierarchical, and demonstrated low ability to change which contributed to the employee survey intervention to fail.

In strong contrast to this stands a case study by Ward (2008). It describes the successful implementation of a survey endeavor at Fujitsu through a consulting firm, whose methodology was “say, stay, strive.” This strategy was aimed at giving employees a voice, giving them incentive to stay with the company, and striving to be better. This fit Fujitsu’s organizational culture well, and top management was very supportive of the survey implementation. The company made an effort to share best practices, and improvements in employee engagement were noted through action planning at the local level.

In summary, only one study specifically examined climate or culture, but we can draw inferences from the descriptions provided by some of the authors. Most likely, this research topic has been given little attention for similar reasons as the neglect of resources. An organization is not likely to collaborate in intervention research when their culture does not allow such efforts.

##### Strategy

None of the studies included in this review contained specific information pertaining to organizational strategy, which poses a large research gap.

#### Human System

##### Employees

Nearly all studies provided descriptions of the employees involved in the studies, as they constituted the participants of their research. Only five studies examined the relevance of group composition and the characteristics of employees participating in feedback meetings. For example, Alderfer and Holbrook (1973) found that group composition was related to the length of time that different topics were discussed. Branch managers mainly discussed authority, control, communication, and conflict, whereas management trainees were mainly concerned with communication, conflict, and careers. Church et al. (2012) examined whether the same pattern of results (i.e., groups that reported action was being taken based on their survey results showed more favorable survey responses over the years) held up for different groups of employees, such as frontline employees, executives, and professionals. Results suggested that frontline managers were more dissatisfied when results were not acted on in comparison to the other two groups, but they were equally satisfied when results were acted upon. Hence, the results held up across different groups of employees.

Gavin and Krois (1983) examined the demographic characteristics of the feedback groups, including employees. For example, younger groups displayed more constructive problem-solving and fewer avoidance behaviors. More highly educated groups spent relatively more time on problem resolution. Nadler et al. (1976, 1980) found differing effects in different departments of a bank (i.e., tellers, financial consultants). The authors concluded that different approaches may be called for in different types of work units that are made up of different kinds of organizational members. Tellers, for example, have little control over their tasks, and higher performance might be less rewarding for them as for financial consultants, who have more autonomy in their tasks. Hence, these groups might have different levels of motivation to engage with the survey feedback data.

Overall, we still know very little about employees’ roles in the survey feedback process and how different individuals might perform and engage in it. Church et al. (2012) already highlighted this gap in the literature almost 10 years ago and noted that different individual predispositions might lead to differing response profiles and subsequently might also affect all following steps (e.g., action planning).

##### Leaders/managers

It is widely accepted throughout the change management literature that leaders and managers play a central role as change agents (Conway and Monks, 2011). Nevertheless, only nine studies gave specific attention to leadership (see Table 1). For example, results suggest that teams led by managers with poor leadership skills potentially benefit most from survey feedback interventions (Solomon, 1976), but managers with low ratings on leadership questions might also be less likely to use the feedback with their units, even though they have the most need to do so (Born and Mathieu, 1996). On the contrary, Conlon and Short (1984) found that managers with higher ratings on an item asking how the manager performs under pressure and how often the manager holds group meetings for communication purposes, were more likely to provide survey feedback to their teams. Even though these items were weak predictors, the authors concluded that supervisors who have preexisting processes in place to discuss work related matters with their teams might be at an advantage to continue such behavior within the scope of the employee survey follow-up. Supervisor ratings also improved after the intervention; more specifically, the intervention had the greatest effect on supervisor ratings in comparison to all other measures (e.g., climate or resources).

Jöns (2000) examined leadership and the type of feedback discussions (with or without a neutral presenter/moderator) as moderators of perceived quality of the feedback meetings and their outcomes. However, the author jointly examined leadership assessments (upward feedback) and employee surveys while acknowledging their close parallels. Self-guided feedback meetings, in comparison to moderated meetings, led to greater improvements in leadership behaviors, but only for groups in which leaders were rated as satisfactory, in comparison to high and very high ratings. Results also suggested that managers improved in their moderation skills over time.

In summary, the results of these studies suggest that managers and leaders play a central role in the employee survey follow-up process, but only few studies examined the characteristics of leaders in-depth to determine which factors contribute to and which might inhibit the employee survey follow-up.

##### Other change agents

Overall, most studies included some kind of change agent or consultant (internal or external) who accompanied the employee survey endeavors in addition to work unit managers. Their involvement in the process differed with regard to intensity, but also with regard to the steps of the employee survey process they supported. However, only three studies specifically examined the role of change agents. For example, Alderfer and Ferriss (1972) found that managers who received support from a consulting team that consisted of insider and outsider consultants were more likely to view the intervention positively and showed more awareness of interpersonal problems. This suggests that it might be beneficial to utilize the expertise of an external consultant who can foster communication across organizational boundaries, but to also have an internal consultant present who understands the specific needs of the team and can evaluate the feasibility of action plans.

We will now provide a few study examples of different levels of change agent involvement from least to most (see Table 1 for an overview). Some studies described no or low involvement of other change agents, which meant that there was, if any, little involvement either during the preparation stage or during the feedback meetings. For example, some studies did not mention any consultant or other change agent supporting the survey feedback process (Björklund et al., 2007; Huebner and Zacher, 2021). Other studies described low involvement of other change agents. For example, in a study of survey feedback in neonatal intensive care units, Baker et al. (2003) reported that team leaders participated in some exercises to foster their understanding of the data, which the study heavily relied on, rather than interpreting the data for managers. However, respondents in several care center units commented that a facilitator or an expert in organizational behavior would have been helpful to support them during the actual feedback meetings in reviewing, interpreting, and highlighting the relevant results and deciding on which topics to target with action planning.

Other studies described a medium level of involvement of consultants, which means that managers received thorough support either during the preparation phase of feedback meetings or during the actual meetings. For example, Born and Mathieu (1996) provided thorough training for supervisors in which they were coached on how to conduct feedback meetings with their teams and how to develop action plans. Then, supervisors were independently responsible for holding the according meetings with their teams. Similarly, Solomon (1976) reported that managers participated in a workshop in which they received the result reports of their teams, received help in interpreting the data, and were guided on how to develop action plans. Subsequently, they held feedback meetings with their teams.

Lastly, some studies described high involvement of other change agents, which means managers received thorough support before and during feedback meetings. For example, in an intervention study by Elo et al. (1998), occupational health physicians and nurses took on active roles by providing consultative support in the face-to-face discussions with work teams and managers, which was furthermore supported by an external researcher–consultant. The occupational health personnel also ensured the continuity of the process and kept participating in the meetings.

Overall, the different grades of change agent involvement and the contrasting results across studies make a definite statement regarding the effectiveness of involving other change agents in the process challenging.

### Output

#### Individual Outcomes

##### Psychological outcomes

The majority of studies (38) provided information about a variety of psychological outcomes following employee survey follow-up processes (see Table 1). For example, a large-scale survey feedback intervention showed improvements in all areas measured by the survey, which mainly related to indicators of workplace culture, such as quality of work life, morale, opportunity for professional growth, and supportive leadership (Jury et al., 2009a,b). Survey feedback has also been shown to lead to increases in readiness to change among executives of the organization (Alderfer and Holbrook, 1973), or improvements in communication, ease in tension in organization, satisfaction, and employee relations (Amba-Rao, 1989). Conlon and Short (1984) reported improved ratings of supervisor behavior, goal clarity, task perceptions, and opportunity for advancement improved during their intervention, whereas at least a medium level of feedback was needed to produce meaningful changes.

However, most results of the studies included in this review were rather mixed. In a short case description by Miles et al. (1969), survey feedback meetings among school staff led to improvements in participant ratings of own openness and collaborative problem-solving, but other improvements, such as in communication, were short-lived. The authors suspected a lack of follow-through regarding the planned actions and a relatively low number of actions generally were the reason that changes did not persist. Björklund et al. (2007) reported that groups with feedback and action plans showed improvements on leadership factors and commitment to the organization, but job demands and control at work did not improve. Adams and Sherwood (1979) reported that one of the intervention groups in a military setting even showed a decline in job satisfaction. However, this group experienced a change in commanders during the intervention, which could have been a possible confound to the study.

Anderzén and Arnetz (2005) found improvements 1 year after their intervention in terms of employee well-being, work-related exhaustion, performance feedback, participatory management, skills development, efficiency, and leadership, but no changes for goal clarity. Church and Oliver (2006) showed that respondents who reported that their survey results had been used for action, rated overall job satisfaction more favorably. Church et al. (2012) followed up on these results with more longitudinal data of the same organization and found that the group that indicated that its survey results had been shared and acted upon, were consistently more favorable raters across all items and across all years.

Another type of psychological outcome is the satisfaction of participants with the feedback process, which most likely influences their motivation to participate in the following feedback sessions. In a study by Peter (1994), the necessary follow-up survey could not be administered to conduct a proper comparison of employee attitudes and turnover intentions before and after the survey feedback intervention due to administrative changes in the organization. However, nursing manages reported high satisfaction with the survey intervention and process in general. Specifically, 75% responded they would want to use the intervention again and 25% indicated that they would probably use it again. In a follow-up study, improvements on job satisfaction could be found for one work unit (Peter et al., 1997). Klein et al. (1971) found that variables such as quality of meetings, the person presenting the information, and the number of meetings influenced how satisfied participants were with the feedback process and data utilization. Also, ratings of feedback quality were higher when meetings were held in person by frontline managers.

In summary, most studies were able to find improvements on a variety of psychosocial outcomes, but results were generally mixed and seemed to differ depending on different factors that could have acted as moderators of the found relationships.

##### Physiological outcomes

Only four studies examined physiological changes following survey feedback interventions, and they were all published in medical and health journals, rather than in industrial and organizational psychology journals. For example, Anderzén and Arnetz (2005) found that improvements in psychosocial work factors were associated with improvements in self-rated health and ratings of quality of sleep. Also, levels of stress-related hormones (i.e., serum triglycerides and serum cholesterol) in blood samples were reduced at an aggregate level after the intervention, and serum testosterone (an important restorative hormone) increased. The authors also measured increased levels of cortisol; low levels of cortisol are indicative of chronic fatigue and burnout. Similarly, Elo et al. (1998) reported reduced mental, but also physical strain for one of the three departments (i.e., finishing department of a factory) in which the survey feedback was implemented.

Eklöf et al. (2004) examined the proportion of workgroup members who reported any workplace modifications with regard to ergonomics (e.g., screen placement, visual conditions, etc.) or with regard to psychosocial aspects (e.g., social support, support from supervisor) following a survey feedback intervention. They found that both outcomes decreased for all feedback groups (i.e., feedback to groups, only to supervisors, only to individual employees) and for the control group. However, the feedback groups positively differed from the control group in that there was less decrease in ergonomic workplace modifications. Importantly, this study did not measure actual modifications or physiological benefits, such as reduced musculoskeletal complaints. The authors also caution that intervention effects could have been inflated or diminished due to a variety of confounds, such as recall bias, control-group effects, and social desirability. This study was followed up on by Eklöf and Hagberg (2006) using the same intervention implementation. The researchers could not find any intervention effects for symptom indicators, such as eye discomfort or musculoskeletal symptoms, which were self-reported as pain or discomfort in neck, shoulder, upper or lower back. There was, however, an improvement in social support measures when feedback was fed back to supervisors only.

In sum, results suggest that physiological benefits can be derived from employee surveys, but results were generally mixed and require further investigation.

#### Organizational Outcomes

Nine studies examined organizational outcomes following survey feedback. For example, Church and Oliver (2006) found that groups that reported action was taken following their surveys showed 50% lower incident rates of accidents on the job and 48% less lost time in days due to accidents. Those groups also showed lower turnover intentions and actual turnover. However, as the turnover data was not longitudinal, causality cannot be inferred. Similarly, Nadler et al. (1976) reported reduced turnover in one of the branches for bank tellers that used the feedback system effectively. Branches that used the feedback system ineffectively even showed a slight increase in turnover. Hautaluoma and Gavin (1975) reported a lower turnover rate for older employees and less absenteeism for blue-collar workers at an organization in which consultants held quite intense survey feedback meetings with staff.

Anderzén and Arnetz (2005) found that as self-rated health ratings increased following the survey intervention, absenteeism decreased. Also, decreased work tempo and improved work climate were related to decreased absenteeism. In contrast, Björklund et al. (2007) could not replicate these findings and did not find decreased sick leave for any of the comparison groups (a group without any feedback, a group with feedback only, and a group with feedback and action planning).

In summary, employee surveys seem to have the potential to lead to improvements in organizational outcomes, such as reduced turnover or absenteeism, but results are mixed and do not seem to hold up in every context.

## Discussion

With an increasing number of organizations that survey their employees (Welbourne, 2016), it is likely that the topic of implementing a proper follow-up process will also continue to gain importance. We reviewed the literature on this topic based on an integrative conceptual model that we developed drawing from Nadler and Tushman’s Congruence Model of Organizational Behavior (1980), Burke and Litwin’s Model of Organizational Performance and Change (1992), and Porras and Robertson’s Change-Based Organizational Framework (1992).

In the following, we summarize the major insights of our review pertaining to each component of the model. By doing so, we answer our research question regarding which variables of our conceptual model have been sufficiently informed by past research and which variables require future research. Based on this discussion, we also provide implications for practice and offer suggestions for future research. Overall, we conclude that research on the employee survey follow-up process has investigated some of the relevant aspects, but large gaps of knowledge remain. Most of the research we reviewed focused on the measurement and achievement of human or organizational outcomes following a survey feedback intervention, which was mostly accomplished with pre/post designs. There were less studies focusing on the process of the employee survey follow-up. Some studies did investigate the process with other research designs, including qualitative interviews with survey practitioners or managers (e.g., Fraser et al., 2009; Wiley, 2012) or by surveying managers who conducted employee follow-up meetings (e.g., Gable et al., 2010). Researchers use longitudinal designs to measure change and to answer questions of causality (Wang et al., 2017). However, there may be also value in other designs that collect cross-sectional or qualitative data.

In this regard, we suggest that more attention should be paid to the organizational actors who drive the employee survey (follow-up) process. In the majority of studies, managers and employees played what seemed a rather passive role in the process in the sense that they were described as attendees to the survey feedback meetings, but their specific characteristics were often not examined. Sometimes, demographic variables (e.g., age, education, marital status) were merely treated as correlates, rather than independent variables (e.g., Peter, 1994). However, these actors are the main organizational stakeholders that drive the process and are mostly affected by it as well. Hence, they play an essential role and should receive more research attention.

Especially the topic of leadership is of great significance. Leaders generally constitute important change agents in organizations (Conway and Monks, 2011) and, accordingly, they play an important role in the employee survey process (Welbourne, 2016). Despite their importance, only few studies examined leadership in this context. However, several studies included in this review mentioned the potential for tension between leaders and subordinates and the resulting lack of psychological safety for participants in the employee survey process (e.g., Alderfer and Holbrook, 1973; Dodd and Pesci, 1977; Baker et al., 2003). This potential for tension between managers/supervisors and subordinates during the employee survey follow-up has not yet been fully explored, but instead was mostly named as a limitation to or challenge of the included studies. In contrast, the issue of reactions to received feedback has received more attention in the upward feedback and 360 degree feedback literature (e.g., Atwater et al., 2000; Atwater and Brett, 2005) and in the performance appraisal literature as well (e.g., Pichler, 2012).

Experts often recommend that an additional change agent should be involved in these other feedback practices to support the recipients of the feedback in the process of understanding the data and using it for developmental purposes. The majority of studies included in this review involved change agents in addition to managers, such as human resource personnel or consultants. However, their level of involvement varied greatly between studies, and differences between groups with and without support by a change agent remain largely unexplored. Some results suggest that some type of support for managers, such as training, may present advantages for the process (Dodd and Pesci, 1977).

Furthermore, other additional research gaps emerged in light of our conceptual model, including the effects of survey items/questionnaires as antecedents to the follow-up tasks. Whereas most studies sufficiently described the surveys they were using, none of them examined the characteristics of the survey as predictors. Related to this, another gap concerns the interpretation of the survey data after it is available to managers. It remains unclear, how the data should best be presented to managers (and also employees), and how much support managers should receive in the process. Another gap concerns the effects of organizational culture/climate, organizational strategy, and the availability of resources on the follow-up process. Almost none of the studies explicitly examined these factors, whereas the results of some case study descriptions suggest that organizational culture and climate could be important to consider (e.g., Swanson and Zuber, 1996; Ward, 2008). As the majority of research described some type of intervention in an organization, it is possible that the above mentioned factors were not explicitly studied because it is likely that they were sufficient when an organization agrees to collaborate in such research. Examining natural settings, for example by retrospectively asking survey practitioners about their experiences in the survey implementation process, could deem useful to further explore these variables.

Generally, this body of literature remains underdeveloped, which stands in contrast to research on more specific workplace interventions that aim to improve worker well-being and job attitudes (e.g., Fox et al., 2021; Solinger et al., 2021). However, other OD interventions are more clearly defined in terms of their goals and, hence, they must be carefully chosen to match the characteristics of the target group (Bowers and Hausser, 1977). For example, a team building intervention might be appropriate to help ameliorate issues pertaining to communication and collaboration in a team (Margulies et al., 1977). There have also been suggestions for interventions targeted at supporting an age-diverse workforce (Truxillo et al., 2015).

In contrast, the employee survey is much less clearly defined as an intervention tool, as the reasons to implement an employee survey vary. Research suggests that, generally, employee surveys are implemented for the purpose of organizational assessment, organizational change (Hartley, 2001), or for improving communication (Kraut, 2006). Also, the assessment of a current situation or current state of organizational culture might be to prepare for the upcoming implementation of change interventions (Hartley, 2001). Hence, the survey is the diagnostic tool that precedes an intervention and is an indicator for the kind of action plans that could be useful. Based on the variety of topics a survey can cover, the types of identified needs to implement a change initiative can be just as versatile and can target different levels of the organization (Falletta and Combs, 2002).

Therefore, examining employee surveys as change tools might be more challenging in comparison to targeted change initiatives with predefined goals. As the following discussion will show, this also hinders a general estimation of employee surveys’ effectiveness in achieving changes. It does, however, argue for the necessity to view the employee survey follow-up in a more differentiated manner, rather as a dichotomous process (i.e., action planning was or was not completed). Different types of interventions following the survey might require different implementation and research approaches than those that are currently applied.

### The Effectiveness of Employee Surveys for Change

Generally, findings were mixed regarding the effectiveness of survey feedback and the employee survey follow-up process. Several studies found benefits for a variety of outcomes, but others could not replicate those findings. As Born and Mathieu (1996) already noted, the quality of change interventions is difficult to gauge between and even within studies, as any given survey feedback intervention is most likely not implemented equally well. For example, Nadler et al. (1980) reported varying levels of intervention implementation between departments regarding the number of meetings held, the people who led discussions, and the extent to which employees got involved in the action planning process. Also, throughout the literature included in this review, some employees received the survey results shortly after the survey (e.g., 2 weeks later; Hautaluoma and Gavin, 1975), and others waited 12 weeks or longer (e.g., Jury et al., 2009a,b). However, most practitioner books and other resources on the topic recommend that results should be available as quickly as possible after survey participation, so that feelings and thoughts during the survey are still present when results are discussed (e.g., Kraut, 2006; Bungard et al., 2007). Also, study durations and the (number of) measurement time points varied greatly from a few weeks or months (e.g., Eklöf et al., 2004; Eklöf and Hagberg, 2006) to several years (e.g., Church et al., 2012). Some results suggested though that the more time participants had to conduct action planning (e.g., 2 years vs. 1 year), the more scores tended to improve (Church and Oliver, 2006; Huebner and Zacher, 2021).

Furthermore, many studies reported issues during the implementation and confounds that could have diluted the results. For example, some researchers reported major restructuring of the organization during the intervention period of 2 years and generally much skepticism and apprehension of the workforce to participate in the survey (Jury et al., 2009a,b). Alderfer and Holbrook (1973) reported that some executives of the company thought that the researchers might have exaggerated the degree of problems that persisted in the company, which indicated a general lack of trust toward the research endeavor.

Related to this issue, we found that the literature provided differing levels of information and descriptions of the actual feedback meetings and developed action plans. Some studies described the intervention with much detail. For example, as one of few studies, Gavin and Krois (1983) examined specifically the topics discussed in feedback meetings and the duration of those discussions. Other studies, on the other hand, reported that feedback meetings were conducted, but the authors admitted that they did not examine how these meetings were conducted (e.g., Björklund et al., 2007; Huebner and Zacher, 2021). Furthermore, very few studies reported or discussed the effect sizes of their interventions, (for exceptions see e.g., La Grange and Geldenhuys, 2008; Huebner and Zacher, 2021). Even though the reporting of standardized effect sizes is widely recommended (Appelbaum et al., 2018), it is oftentimes neglected in research, which hinders the ability to draw interferential conclusions from the study results (Kelley and Preacher, 2012).

In summary, we conclude that such a great variety in quality of implementation and descriptions of the interventions limits their comparability and the conclusions that can be drawn from this research. Nevertheless, the majority of studies were able to find positive effects on some outcomes, which suggests that employee surveys can have beneficial effects in organizations when used to implement a proper follow-up. These conclusions should be viewed with caution though, as results might have been affected by publication bias because null results tend to not get published (Landis et al., 2014).

### Implications for Practice

Even though there are many books on the topic, the employee survey process remains challenging, and many organizations fail to harvest the full benefits of this common human resource practice (Brown, 2021). Depending on the organization, different change agents or organizational actors might be responsible for the implementation of the process (e.g., internal or external consultants/survey practitioners, human resource administrators, or managers), which creates ambiguity and the difficulty of finding the best implementation strategy. It is important for the responsible organizational actors to acknowledge that there is no “one size fits all” approach to employee surveys and their follow-up. Different organizations will thrive with different implementation models, depending on their culture, work environment, and staff.

Nevertheless, some recommendations can be offered based on this review. It seems to be most effective to not only provide survey feedback data, but to also make sure that actual action planning takes place (Bowers, 1973; Björklund et al., 2007; Church et al., 2012). Also, it is beneficial when the questionnaire fits the organization, and the items are actionable for managers and their teams (Church et al., 2012). Managers should be properly involved in the follow-up process, as they are the key change agents who must drive the implementation of action plans (Mann and Likert, 1952; Welbourne, 2016). However, it is also important that managers receive the necessary tools to do the job. These tools include training, sufficient time, support from top management, and other necessary resources (Wiley, 2012). The involvement of other change agents, such as consultants who help analyze the data, can be beneficial, but managers should not create the habit of relying too heavily on such resources. They should rather be enabled and trained to understand and utilize the data self-reliantly in collaboration with their teams. On that note, other supporting tools, such as SKDA can be useful aids, but they do not exempt managers from properly understanding the data. Supporting change agents might also be helpful in situations where there is much tension between managers and subordinates, which could potentially inhibit fruitful feedback discussions. Lastly, high involvement of all stakeholders seems to most beneficial as it creates accountability and a deeper understanding and acceptance of the actions following the survey (Mann and Likert, 1952).

Whereas following this set of recommendations will not guarantee a perfect employee survey follow-up implementation, we believe it can help. Implementing employee surveys is costly, and designing a useful follow-up can support organizations in getting the most out of their investment. Benefits can manifest as improvements in employee attitudes, physiological outcomes, and even organizational factors, as for example reduced turnover. Consequently, organizations should evaluate how ready their workforce is to master the employee survey follow-up. In the beginning, managers might require more support, but as they become more acquainted and comfortable with the process, and they have been enabled to function as active change agents in the organization, they might need less resources as support.

### Limitations and Suggestions for Future Research

There are a few limitations to this systematic review worth noting. One limitation includes our method of searching for relevant literature in Google Scholar. One of this database’s shortcomings is that the search algorithm changes every day, making the search not completely replicable at a later point in time (Bramer et al., 2016). Also, Google Scholar has low recall capabilities (only the first 1000 results are viewable), which is why it is preferable to also search in an additional database (Bramer et al., 2016).

Another limitation is the exclusion of gray literature. As we only included studies published in peer-reviewed journals and edited books, the overall results might be subject to publication bias as null results tend to not get published (Landis et al., 2014). Hence, as previously mentioned, the results of this review regarding the effectiveness of employee surveys for the purpose of OD should be viewed with caution.

Overall, drawing from other areas of industrial and organizational psychology, as for example the literature on leadership, teams, employee voice, and engagement could prove useful to examine the variables of the model that have not been sufficiently explored. For example, research on leadership suggests that different kinds of leadership behaviors contribute to the job performance of employees, but that such effects also depend on certain characteristics of employees (Breevaart et al., 2016). Hence, leadership is an important variable that deserves more research attention, which could be accomplished by the application of leadership theories. Group voice climate also seems to be related to perceptions of leadership and group performance (Frazier and Bowler, 2015), but as can be seen in Table 1, culture and climate have not been fully explored as predictors or moderators of the employee survey process. Hence, we recommend cross-cultural examinations of post-survey practices. The alignment between company and employee survey strategy could also be crucial for this process, and we suggest examining such by conducting research in which the degree of alignment is measured. We also suggest that external factors should be examined in this research context. For example, the type of industry that the feedback meetings are held in could influence meeting effectiveness because action planning could be more or less influential, depending on industry-bound work environments.

Furthermore, we believe that research on the post-survey process would benefit from integrating and drawing from survey research, as for example research pertaining to survey modes (e.g., Borg and Zuell, 2012; Mueller et al., 2014) or questionnaire design and development (e.g., Roberson and Sundstrom, 1990; Alden, 2007). The survey itself should be considered an antecedent of the follow-up, as the type of data and data format could influence how the follow-up is carried out. Lastly, most studies included additional change agents who were involved in the survey feedback process, but future research should investigate these organizational actors in more depth. For example, qualitative data from experienced change agents could render important findings in regards to factors that inhibit or enhance the process from their perspective.

Overall, this body of literature provides much opportunity for the further integration of adjacent research areas, including other areas within industrial and organizational psychology, and for more theory-driven research. Whereas most records were published in industrial and organizational psychology journals, we also found some studies in journals of other disciplines, such as education or medicine (see Appendix). We propose that research in this area would benefit from more cross-disciplinary approaches. For example, research regarding physiological outcomes of survey feedback interventions might require the expertise of medical professionals.

Other disciplines, such as social psychology, could also provide useful insights for this research area. For example, the theory of planned behavior (Ajzen, 2002) or control theory (Carver and Scheier, 1982) could help explain certain behaviors during the employee survey follow-up discussions and render important findings for these processes. Applying other behavioral theories to the employee survey context could also put forth important findings, as for example goal setting theory (Locke and Latham, 1990) or fundamental attribution error (Ross, 1977).

Due to the applied nature of employee survey research, using experimental designs, specifically randomized controlled trials, can be challenging. Nevertheless, we believe this would be useful to examine the factors named above in more detail. Such designs could aid in systematically testing different process variables that are relevant for the employee survey follow-up. Also, examining the differing time intervals between the survey, receiving feedback, and action planning, should be examined, especially in light of the growing popularity of pulse surveys (Welbourne, 2016). However, natural experiments can also render important findings regarding for example resources, as deficits in such might not become exposed unless natural settings are studied.

Overall, research on this topic has seemed to almost come to a halt. Out of 53 studies, 47 (∼90%) were published before 2010 – over 10 years ago. However, with increasing digitalization and the influx of new tools and ways of collaborating at the workplace, we require more research in this area to meet the newly emerging needs of organizations. This is especially relevant in light of the ongoing COVID-19 pandemic, which has started to change everyday life at work (Allen et al., 2020; Rudolph et al., 2021).

## Conclusion

Even though leaders can talk to their subordinates daily and on a regular basis, the employee survey and its follow-up remains an important communication forum for them. Generally, the results of this review suggest that the employee survey follow-up can lead to a variety of benefits for and improvements in organizations, but we have not sufficiently explored all factors that can support or inhibit this process. The literature yields many important findings for practitioners regarding the implementation and effectiveness of the process, but some research gaps remain. Hence, future research in this area should focus more on the relevant process variables and organizational actors involved, especially leaders who function as the main change agents in this data-based approach to OD.

## Data Availability Statement

The original contributions presented in the study are included in the article/Supplementary Material, further inquiries can be directed to the corresponding author/s.

## Author Contributions

L-AH and HZ contributed to conception and planning of the systematic review. L-AH performed the literature searches, organized the data, and wrote the first draft of the manuscript. Both authors contributed to manuscript revision, read, and approved the submitted version.

## Author Disclaimer

The results, opinions, and conclusions expressed in this paper are not necessarily those of Volkswagen Aktiengesellschaft.

## Conflict of Interest

L-AH was employed by company Volkswagen AG. The remaining authors declare that the research was conducted in the absence of any commercial or financial relationships that could be construed as a potential conflict of interest.

## Publisher’s Note

All claims expressed in this article are solely those of the authors and do not necessarily represent those of their affiliated organizations, or those of the publisher, the editors and the reviewers. Any product that may be evaluated in this article, or claim that may be made by its manufacturer, is not guaranteed or endorsed by the publisher.

## Footnotes

1 The protocol can be found here: https://osf.io/y5be9/?view_only=f0ca973da2334db1b504291318b7c402

2 The list of all references can be found here: https://osf.io/y5be9/?view_only=f0ca973da2334db1b504291318b7c402

## References

[B1] Adams J. Sherwood J. J. (1979). An evaluation of organizational effectiveness: an appraisal of how army internal consultants use survey feedback in a military setting. *Group Organ. Stud.* 4 170–182. 10.1177/105960117900400205

[B2] Aguinis H. Gottfredson R. K. Joo H. (2012). Delivering effective performance feedback: the strengths-based approach. *Bus. Horiz.* 55 105–111. 10.1016/j.bushor.2011.10.004

[B3] Ajzen I. (2002). Residual effects of past behavior on later behavior: habituation and reasoned action perspectives. *Pers. Soc. Psychol. Rev.* 6 107–122. 10.1207/S15327957PSPR0602_02

[B4] Alden J. (2007). Surveying attitudes: questionnaires versus opinionnaires. *Perform. Improv.* 46 42–48. 10.1002/pfi.141

[B5] Alderfer C. P. Ferriss R. (1972). “Understanding the impact of survey feedback,” in *The Social Technology of Organization Development*, eds Burke W. W. Hornstein H. A. (Bethel, ME: National Training Laboratories), 234–243.

[B6] Alderfer C. P. Holbrook J. (1973). A new design for survey feedback. *Educ. Urban Soc.* 5 437–464. 10.1177/001312457300500405

[B7] Allen J. B. Jain S. Church A. H. (2020). Using a pulse survey approach to drive organizational change. *Organ. Dev. Rev.* 52 62–68.

[B8] Amba-Rao S. (1989). Survey feedback in a small manufacturing firm: an application. *Organ. Dev. J.* 7 92–100.

[B9] Anderzén I. Arnetz B. B. (2005). The impact of a prospective survey-based workplace intervention program on employee health, biologic stress markers, and organizational productivity. *J. Occup. Environ. Med.* 47 671–682. 10.1097/01.jom.0000167259.03247.1e 16010194

[B10] Appelbaum M. Cooper H. Kline R. B. Mayo-Wilson E. Nezu A. M. Rao S. M. (2018). Journal article reporting standards for quantitative research in psychology: the APA publications and communications board task force report. *Am. Psychol.* 73 3–25. 10.1037/amp0000191 29345484

[B11] Atwater L. E. Brett J. F. (2005). Antecedents and consequences of reactions to developmental 360^°^ feedback. *J. Vocat. Behav.* 66 532–548. 10.1016/j.jvb.2004.05.003

[B12] Atwater L. E. Brett J. F. Charles A. C. (2007). Multisource feedback: lessons learned and implications for practice. *Hum. Resour. Manag.* 46 285–307. 10.1002/hrm.20161

[B13] Atwater L. E. Waldman D. A. Atwater D. Cartier P. (2000). An upward feedback field experiment: supervisors’ cynicism, reactions, and commitment to subordinates. *Pers. Psychol.* 53 275–297. 10.1111/j.1744-6570.2000.tb00202.x

[B14] Baker R. G. King H. MacDonald J. L. Horbar J. D. (2003). Using organizational assessment surveys for improvement in neonatal intensive care. *Pediatrics* 111 419–425. 10.1542/peds.111.SE1.e419 12671161

[B15] Beer M. Walton A. E. (1987). Organization change and development. *Annu. Rev. Psychol.* 38 339–367. 10.1146/annurev.ps.38.020187.002011

[B16] Besieux T. (2017). Why I hate feedback: anchoring effective feedback within organizations. *Bus. Horiz.* 60 435–439. 10.1016/j.bushor.2017.03.001

[B17] Björklund C. Grahn A. Jensen I. Bergström G. (2007). Does survey feedback enhance the psychosocial work environment and decrease sick leave? *Eur. J. Work Organ. Psychol.* 16 76–93. 10.1080/13594320601112169

[B18] Bordia P. Restubog S. L. D. Jimmieson N. L. Irmer B. E. (2011). Haunted by the past: effects of poor change management history on employee attitudes and turnover. *Group Organ. Manag.* 36 191–222. 10.1177/1059601110392990

[B19] Borg I. Zuell C. (2012). Write-in comments in employee surveys. *Int. J. Manpow.* 33 206–220. 10.1108/01437721211225453

[B20] Born D. Mathieu J. (1996). Differential effects of survey-guided feedback. *Group Organ. Manag.* 21 388–403. 10.1177/1059601196214002

[B21] Bowers D. G. (1973). OD techniques and their results in 23 organizations: the Michigan ICL study. *J. Appl. Behav. Sci.* 9 21–43. 10.1177/002188637300900103

[B22] Bowers D. G. Hausser D. L. (1977). Work group types and intervention effects in organizational development. *Adm. Sci. Q.* 22 76–94. 10.2307/2391747

[B23] Bracken D. W. Timmreck C. W. Fleenor J. W. Lynn S. (2001). 360 feedback from another angle. *Hum. Resour. Manag.* 40 3–20. 10.1002/hrm.4012

[B24] Bramer W. M. Giustini D. Kramer B. M. R. (2016). Comparing the coverage, recall, and precision of searches for 120 systematic reviews in Embase, MEDLINE, and google scholar: a prospective study. *Syst. Rev.* 5 1–7. 10.1186/s13643-016-0215-7 26932789 PMC4772334

[B25] Breevaart K. Bakker A. B. Demerouti E. Derks D. (2016). Who takes the lead? A multi-source diary study on leadership, work engagement, and job performance. *J. Organ. Behav.* 37 309–325. 10.1002/job.2041

[B26] Brown L. D. (1972). “Research action”: organizational feedback, understanding, and change. *J. Appl. Behav. Sci.* 8 697–711. 10.1177/002188637200800606

[B27] Brown M. I. (2021). Does action planning create more harm than good? Common challenges in the practice of action planning after employee surveys. *J. Appl. Behav. Sci.* 10.1177/00218863211007555 [Epub ahead of print].

[B28] Bungard W. Müller K. Niethammer C. (eds). (2007). *Mitarbeiterbefragung - Was Dann …? MAB und Folgeprozesse Erfolgreich Gestalten [Employee surveys – and then what …? Successfully Designing Employee Surveys and the Follow-Up Process].* Heidelberg: Springer Medizin Verlag, 10.1007/978-3-540-47841-6

[B29] Burke W. W. Coruzzi C. A. Church A. H. (1996). “The organizational survey as an intervention for change,” in *Organizational Surveys: Tools for Assessment and Change*, ed. Kraut A. I. (San Francisco, CA: Jossey-Bass), 41–66.

[B30] Burke W. W. Litwin G. H. (1992). A causal model of organizational performance and change. *J. Manag. Dev.* 18 523–545. 10.1177/014920639201800306

[B31] Carver C. S. Scheier M. F. (1982). Control theory: a useful conceptual framework for personality–social, clinical, and health psychology. *Psychol. Bull.* 92 111–135. 10.1037/0033-2909.92.1.111 7134324

[B32] Cattermole G. Johnson J. Roberts K. (2013). Employee engagement welcomes the dawn of an empowerment culture. *Strateg. HR Rev.* 12 250–254. 10.1108/shr-04-2013-0039

[B33] Chesler M. Flanders M. (1967). Resistance to research and research utilization: the death and life of a feedback attempt. *J. Appl. Behav. Sci.* 3 469–487. 10.1177/002188636700300403

[B34] Church A. H. Golay L. M. Rotolo C. T. Tuller M. D. Shull A. C. Desrosiers E. I. (2012). “Without effort there can be no change: reexamining the impact of survey feedback and action planning on employee attitudes,” in *Research in Organizational Change and Development*, Vol. 20 eds Woodman R. W. Pasmore W. A. Rami Shani A. B. (Bingley: Emerald Group Publishing), 223–264.

[B35] Church A. H. Margiloff A. Coruzzi C. (1995). Using surveys for change: an applied example in a pharmaceuticals organization. *Leadersh. Organ. Dev. J.* 16 3–11. 10.1108/01437739510089049

[B36] Church A. H. Oliver D. H. (2006). “The importance of taking action, not just sharing survey feedback” in *Getting Action From Organizational Surveys: New Concepts, Technologies, and Applications*, ed. Kraut A. I. (San Francisco, CA: Jossey-Bass), 102–130.

[B37] Church A. H. Waclawski J. (2017). *Designing and Using Organizational Surveys.* London: Routledge.

[B38] Conlon E. J. Short L. O. (1984). An empirical examination of survey feedback as an organizational change device. *Group Organ. Stud.* 9 399–416. 10.5465/ambpp.1983.4976350

[B39] Conway E. Monks K. (2011). Change from below: the role of middle managers in mediating paradoxical change. *Hum. Resour. Manag. J.* 21 190–203. 10.1111/j.1748-8583.2010.00135.x

[B40] Costello J. Clarke C. Gravely G. D’Agostino-Rose D. Puopolo R. (2011). Working together to build a respectful workplace: transforming OR culture. *AORN J.* 93 115–126. 10.1016/j.aorn.2010.05.030 21193084

[B41] Cucina J. M. Walmsey P. T. Gast I. F. Martin N. R. Curtin P. (2017). Survey key driver analysis: are we driving down the right road? *Ind. Organ. Psychol.* 10 234–257. 10.1017/iop.2016.97

[B42] Daniels K. (2018). Guidance on conducting and reviewing systematic reviews (and meta-analyses) in work and organizational psychology. *Eur. J. Work Organ. Psychol.* 28 1–10. 10.1080/1359432X.2018.1547708

[B43] De Waal A. (2014). The employee survey: benefits, problems in practice, and the relation with the high performance organization. *Strateg. HR Rev.* 13 227–232. 10.1108/SHR-07-2014-0041

[B44] Demerouti E. Bakker A. B. Nachreiner F. Schaufeli W. B. (2001). The job demands-resources model of burnout. *J. Appl. Psychol.* 86 499–512. 10.1037//0021-9010.86.3.499 11419809

[B45] DeNisi A. S. Kluger A. N. (2000). Feedback effectiveness: can 360-degree appraisals be improved? *Acad. Manag. Exec.* 14 129–139. 10.5465/ame.2000.2909845

[B46] Dillman D. A. Smyth J. D. Christian L. M. (2014). *Internet, Phone, Mail, and Mixed-Mode Surveys: The Tailored Design Method*, 4th Edn. Hoboken, NJ: John Wiley & Sons, Inc.

[B47] Dodd W. E. Pesci M. L. (1977). Managing morale through survey feedback. *Bus. Horiz.* 20 36–45. 10.1016/0007-6813(77)90069-6

[B48] Duncan R. (1979). What is the right organization structure? Decision tree analysis provides the answer. *Organ. Dyn.* 7 59–80. 10.1016/0090-2616(79)90027-5

[B49] Eklöf M. Hagberg M. (2006). Are simple feedback interventions involving workplace data associated with better working environment and health? A cluster randomized controlled study among Swedish VDU workers. *Appl. Ergon.* 37 201–210. 10.1016/j.apergo.2005.04.003 15982632

[B50] Eklöf M. Hagberg M. Toomingas A. Tornqvist E. W. (2004). Feedback of workplace data to individual workers, workgroups or supervisors as a way to stimulate working environment activity: a cluster randomized controlled study. *Int. Arch. Occup. Environ. Health* 77 505–514. 10.1007/s00420-004-0531-4 15558302

[B51] Elo A.-L. Leppänen A. Sillanpää P. (1998). Applicability of survey feedback for an occupational health method in stress management. *Occup. Med.* 48 181–188. 10.1093/occmed/48.3.181 9659728

[B52] Falletta S. V. Combs W. (2002). “Surveys as a tool for organization development and change,” in *Organization Development: A Data-Driven Approach to Organizational Change*, eds Waclawski J. Church A. H. (Hoboken, NJ: John Wiley & Sons), 78–101.

[B53] Feather K. (2008). Helping HR to measure up: arming the “soft” function with hard metrics. *Strateg. HR Rev.* 7 28–33. 10.1108/14754390810847531

[B54] Fedor D. B. Eder R. W. Buckley M. R. (1989). The contributory effects of supervisor intentions on subordinate feedback responses. *Organ. Behav. Hum. Decis. Process.* 44 396–414. 10.1016/0749-5978(89)90016-2

[B55] Foster C. A. Law M. R. F. (2006). How many perspectives provide a compass? Differentiating 360-degree feedback and multi-source feedback. *Int. J. Sel. Assess.* 14 288–291. 10.1111/j.1468-2389.2006.00347.x

[B56] Fox K. E. Johnson S. T. Berkman L. F. Sianoja M. Soh Y. Kubzansky L. D. (2021). Organisational- and group-level workplace interventions and their effect on multiple domains of worker well-being: a systematic review. *Work Stress.* 10.1080/02678373.2021.1969476 [Epub ahead of print].

[B57] Fraser K. J. Leach D. J. Webb S. (2009). Employee surveys: guidance to facilitate effective action. *Eur. Work Organ. Psychol. Pract.* 3 16–23.

[B58] Frazier M. L. Bowler W. M. (2015). Voice climate, supervisor undermining, and work outcomes. *J. Manag.* 41 841–863. 10.1177/0149206311434533

[B59] Friedlander F. Brown L. D. (1974). Organization development. *Annu. Rev. Psychol.* 1 313–341. 10.1146/annurev.ps.25.020174.001525

[B60] Gable S. A. Chyung S. Y. Marker A. Winiecki D. (2010). How should organizational leaders use employee engagement survey data? *Perform. Improv.* 49 17–25. 10.1002/pfi.20140

[B61] Gavin J. F. Krois P. A. (1983). Content and process of survey feedback sessions and their relation to survey responses: an initial study. *Group Organ. Stud.* 8 221–247. 10.1177/105960118300800208

[B62] Gavin J. F. McPhail S. M. (1978). Intervention and evaluation: a proactive team approach to OD. *J. Appl. Behav. Sci.* 14 175–194. 10.1177/002188637801400203

[B63] Gehlbach H. Robinson C. D. Finefter-Rosenbluh I. Benshoof C. Schneider J. (2018). Questionnaires as interventions: can taking a survey increase teachers’ openness to student feedback surveys? *Educ. Psychol.* 38 350–367. 10.1080/01443410.2017.1349876

[B64] Goldberg B. Gordon G. G. (1978). Designing attitude surveys for management action. *Pers. J.* 57 546–549.

[B65] Griffin M. A. Hart P. M. Wilson-Evered E. (2000). “Using employee opinion surveys to improve organizational health,” in *Healthy and Productive Work: An International Perspective*, eds Murphy L. R. Cooper C. L. (London: Taylor & Francis), 15–36.

[B66] Guzzo R. A. Jette R. D. Katzell R. A. (1985). The effects of psychologically based intervention programs on worker productivity: a meta-analysis. *Pers. Psychol.* 38 275–291. 10.1111/j.1744-6570.1985.tb00547.x

[B67] Hand H. H. Estafen B. D. Sims H. P. Jr. (1975). How effective is data survey and feedback as a technique of organization development? An experiment. *J. Appl. Behav. Sci.* 11 333–347. 10.1177/002188637501100306

[B68] Hannum K. M. (2007). Measurement equivalence of 360^°^ assessment data: are different raters rating the same constructs. *Int. J. Sel. Assess.* 27 293–301. 10.1111/j.1468-2389.2007.00389.x

[B69] Hartley J. (2001). Employee surveys–strategic aid or hand-grenade for organisational and cultural change? *Int. J. Public Sect. Manag.* 14 184–204. 10.1108/09513550110390846

[B70] Hautaluoma J. E. Gavin J. F. (1975). Effects of organizational diagnosis and intervention on blue-collar “blues”. *J. Appl. Behav. Sci.* 11 475–496. 10.1177/002188637501100408

[B71] Hopkins D. (1982). Survey feedback as an organisation development intervention in educational settings: a review. *Educ. Manag. Adm.* 10 203–215. 10.1177/174114328201000304

[B72] Huebner L.-A. Zacher H. (2021). Effects of action planning after employee surveys. *J. Pers. Psychol.* 10.1027/1866-5888/a000285 Advance online publication 37379784

[B73] Hyland P. K. Woo V. A. Reeves D. W. Garrad L. (2017). In defense of responsible survey key driver analysis. *Ind. Organ. Psychol.* 10 277–283. 10.1017/iop.2017.19

[B74] Ilgen D. R. Fisher C. D. Taylor S. M. (1979). Consequences of individual feedback on behavior in organizations. *J. Appl. Psychol.* 64 349–371. 10.1037//0021-9010.64.4.349

[B75] Jacoby J. Mazursky D. Troutman T. (1984). When feedback is ignored: disutility of outcome feedback. *J. Appl. Psychol.* 69 531–545. 10.1037/0021-9010.69.3.531

[B76] Johnson J. W. (2017). Best practice recommendations for conducting key driver analysis. *Ind. Organ. Psychol.* 10 298–305. 10.1017/iop.2017.22

[B77] Jöns I. (2000). “Supervisors as moderators of survey feedback and change processes in teams,” in *Innovative Theories, Tools and Practices in Work and Organizational Psychology*, eds Vartiainen M. Avallone F. Anderson N. (Toronto, ON: Hogrefe & Huber), 155–171.

[B78] Jury C. Goh H. E. Olsen S. P. Elston J. Phillips J. (2009a). Actions and results from the Queensland health “better workplaces” staff opinion survey. *Aust. Health Rev.* 33 371–376. 10.1071/AH090371 20128751

[B79] Jury C. Machin M. A. Phillips J. Goh H. E. Olsen S. P. Patrick J. (2009b). Developing and implementing an action-oriented staff survey: Queensland health and the “better workplaces” initiative. *Australian Health Review* 33 365–370. 10.1071/AH090365 20128750

[B80] Katz D. Kahn R. L. (eds). (1978). *The Social Psychology of Organizations*, Vol. 2. New York, NY: Wiley.

[B81] Keiser N. L. Payne S. C. (2019). Are employee surveys biased? Impression management as a response bias in workplace safety constructs. *Saf. Sci.* 118 453–465. 10.1016/j.ssci.2019.05.051

[B82] Kelley K. Preacher K. J. (2012). On effect size. Psychol. *Methods* 17, 137–152. 10.1037/a0028086 22545595

[B83] Klein C. Synovec R. Zhang H. Lovato C. Howes J. Feinzig S. (2017). Survey key driver analysis: perhaps the right question is, “are we there yet?”. *Ind. Organ. Psychol.* 10 283–290. 10.1017/iop.2017.20

[B84] Klein S. Kraut A. Wolfson A. (1971). Employee reactions to attitude survey feedback: a study of the impact of structure and process. *Adm. Sci. Q.* 16 497–514. 10.2307/2391769

[B85] Kluger A. N. DeNisi A. (1996). The effects of feedback interventions on performance: a historical review, a meta-analysis, and a preliminary feedback intervention theory. *Psychol. Bull.* 119 254–284. 10.1037//0033-2909.119.2.254

[B86] Knapp P. Mujtaba B. (2010). Designing, administering, and utilizing an employee attitude survey. *J. Behav. Stud. Bus.* 2 1–14.

[B87] Kraut A. I. (ed.) (2006). *Getting Action from Organizational Surveys: New Concepts, Technologies, and Applications.* San Francisco, CA: Jossey-Bass.

[B88] La Grange A. Geldenhuys D. J. (2008). The impact of feedback on changing organisational culture. *South. Afr. Bus. Rev.* 12 37–66.

[B89] Landis R. S. James L. R. Lance C. E. Pierce C. A. Rogelberg S. G. (2014). When is nothing something? Editorial for the null results special issue of journal of business and psychology. *J. Bus. Psychol.* 29 163–167. 10.1007/s10869-014-9347-8

[B90] Linke R. (2018). *Mitarbeiterbefragungen Optimieren: Von der Befragung zum Wirksamen Management-Instrument [Optimizing Employee Surveys: From the Survey to the Effective Management Tool].* Wiesbaden: Springer Gabler.

[B91] Locke E. A. Latham G. P. (1990). *A Theory of Goal Setting & Task Performance.* Englewood Cliffs, NJ: Prentice Hall, Inc.

[B92] Macey W. H. Daum D. L. (2017). SKDA in context. *Ind. Organ. Psychol.* 10 268–277. 10.1017/iop.2017.18

[B93] Macey W. H. Fink A. A. (2020). “Surveys and sensing: realizing the promise of listening to employees,” in *Employee Surveys and Sensing: Challenges and Opportunities*, eds Macey W. H. Fink A. A. (Oxford: Oxford University Press), 1–22.

[B94] Mann F. Likert R. (1952). The need for research on the communication of research results. *Hum. Organ.* 11 15–19.

[B95] Margulies N. Wright P. L. Scholl R. W. (1977). Organization development techniques: their impact on change. *Group Organ. Stud.* 2 428–448. 10.1177/105960117700200405

[B96] Miles M. B. Hornstein H. A. Callahan D. M. Calder P. H. Schiavo S. R. (1969). “The consequence of survey feedback: theory and evaluation,” in *The Planning of Change*, 2nd Edn, eds Bennis W. G. Benne K. D. Chin R. (New York, NY: Holt, Rinehart and Winston), 457–468.

[B97] Moher D. Shamseer L. Clarke M. Ghersi D. Liberati A. Petticrew M. (2015). Preferred reporting items for systematic review and meta-analysis protocols (PRISMA-P) 2015 statement. *Syst. Rev.* 4 1–9. 10.1186/2046-4053-4-1 25554246 PMC4320440

[B98] Mueller K. Straatmann T. Hattrup K. Jochum M. (2014). Effects of personalized versus generic implementation of an intra-organizational online survey on psychological anonymity and response behavior: a field experiment. *J. Bus. Psychol.* 29 169–181. 10.1007/s10869-012-9262-9

[B99] Nadler D. A. (1976). The use of feedback for organizational change: promises and pitfalls. *Group Organ. Stud.* 1 177–186. 10.1177/105960117600100205

[B100] Nadler D. A. (1979). The effects of feedback on task group behavior: a review of the experimental research. *Organ. Behav. Hum. Perform.* 23 309–338. 10.1016/0030-5073(79)90001-1

[B101] Nadler D. A. (1980). “Using organizational assessment data for planned organizational change,” in *Organizational Assessment: Perspectives on the Measurement of Organizational Behavior and the Quality of Work Life*, eds Lawler E. E. III Nadler D. Cammann C. (New York, NY: Wiley), 72–90.

[B102] Nadler D. A. (1981). Managing organizational change: an integrative perspective. *J. Appl. Behav. Sci.* 17 191–211. 10.1177/002188638101700205

[B103] Nadler D. A. Cammann C. Mirvis P. H. (1980). Developing a feedback system for work units: a field experiment in structural change. *J. Appl. Behav. Sci.* 16 41–62. 10.1177/002188638001600105

[B104] Nadler D. A. Mirvis P. Cammann C. (1976). The ongoing feedback system: experimenting with a new managerial tool. *Organ. Dyn.* 4 63–80. 10.1016/0090-2616(76)90045-0

[B105] Nadler D. A. Tushman M. L. (1980). A model for diagnosing organizational behavior. *Organ. Dyn.* 9 35–51. 10.1016/0090-2616(80)90039-x

[B106] Neuman G. A. Edwards J. E. Raju N. S. (1989). Organizational development interventions: a meta-analysis of their effects on satisfaction and other attitudes. *Pers. Psychol.* 42 461–489. 10.1111/j.1744-6570.1989.tb00665.x

[B107] Nowack K. M. Mashihi S. (2012). Evidence-based answers to 15 questions about leveraging 360-degree feedback. *Consult. Psychol. J. Pract. Res.* 64 157–182. 10.1037/a0030011

[B108] Ostroff C. (1992). The relationship between satisfaction, attitudes, and performance: an organizational level analysis. *J. Appl. Psychol.* 77 963–974. 10.1037//0021-9010.77.6.963

[B109] Peter M. A. (1994). Making the hidden obvious. Management education through survey feedback. *J. Nurs. Adm.* 24 13–19. 10.1097/00005110-199406000-00006 8006697

[B110] Peter M. A. Lytle K. S. Swearengen P. (1997). Feedback to nurse managers about staff nurses’ perceptions of their jobs. *Semin. Nurse Manag.* 5 209–216. 9460480

[B111] Pichler S. (2012). The social context of performance appraisal and appraisal reactions: a meta-analysis. *Hum. Resour. Manag.* 51 709–732. 10.1002/hrm.21499

[B112] Porras J. I. (1979). The comparative impact of different OD techniques and intervention intensities. *J. Appl. Behav. Sci.* 15 156–178. 10.1177/002188637901500204

[B113] Porras J. I. Berg P. O. (1978). The impact of organization development. *Acad. Manag. Rev.* 3 249–266. 10.2307/257666

[B114] Porras J. I. Robertson P. J. (1992). “Organizational development: theory, practice, and research,” in *Handbook of Industrial and Organizational Psychology*, 3rd Edn, eds Dunnette M. D. Hough L. M. (Palo Alto, CA: Consulting Psychologists Press), 719–822.

[B115] Roberson M. Sundstrom E. (1990). Questionnaire design, return dates, and response favorableness in an employee attitude questionnaire. *J. Appl. Psychol.* 75 354–357. 10.1037/0021-9010.75.3.354

[B116] Rogelberg S. G. Luong A. Sederburg M. E. Cristol D. S. (2000). Employee attitude surveys: examining the attitudes of noncompliant employees. *J. Appl. Psychol.* 85 284–293. 10.1037/0021-9010.85.2.284 10783544

[B117] Rollins T. (1994). Turning employee survey results into high-impact improvements. *Employ. Relat. Today* 21 35–44. 10.1002/ert.3910210105

[B118] Ross L. (1977). “The intuitive psychologist and his shortcomings: distortions in the attribution process,” in *Advances in Experimental Social Psychology*, 10th Edn, ed. Berkowitz L. (New York, NY: Academic Press), 173–220.

[B119] Rotolo C. T. Price B. A. Fleck C. R. Smoak V. J. Jean V. (2017). Survey key driver analysis: our GPS to navigating employee attitudes. *Ind. Organ. Psychol. Perspect. Sci. Pract.* 10 306–313. 10.1017/iop.2017.23

[B120] Rudolph C. W. Allan B. Clark M. Hertel G. Hirschi A. Kunze F. (2021). Pandemics: implications for research and practice in industrial and organizational psychology. *Indus. Organ. Psychol.* 14 1–35. 10.1017/iop.2020.48

[B121] Scherbaum C. A. Black J. Weiner S. P. (2017). With the right map, survey key driver analysis can help get organizations to the right destination. *Indus. Organ. Psychol.* 10 290–298. 10.1017/iop.2017.21

[B122] Siddaway A. P. Wood A. M. Hedges L. V. (2019). How to do a systematic review: a best practice guide for conducting and reporting narrative reviews, meta-analyses, and meta-syntheses. *Annu. Rev. Psychol.* 70 747–770. 10.1146/annurev-psych-010418-102803 30089228

[B123] Solinger O. N. Joireman J. Vantillborgh T. Balliet D. P. (2021). Change in unit-level job attitudes following strategic interventions: a meta-analysis of longitudinal studies. *J. Organ. Behav.* 42 964–986. 10.1002/job.2523

[B124] Solomon R. J. (1976). An examination of the relationship between a survey feedback O.D. technique and the work environment. *Pers. Psychol.* 29 583–594. 10.1111/j.1744-6570.1976.tb02081.x

[B125] Sonnentag S. Frese M. (2002). “Performance concepts and performance theory,” in *Psychological Management of Individual Performance*, ed. Sonnentag S. (Chichester: John Wiley & Sons), 3–25.

[B126] Sugheir J. Coco M. Kaupins G. (2011). Perceptions of organizational survey within employee engagement efforts. *Int. J. Bus. Public Adm.* 8 48–61.

[B127] Swanson R. A. Zuber J. A. (1996). A case study of a failed organization development intervention rooted in the employee survey process. *Perform. Improv. Q.* 9 42–56. 10.1111/j.1937-8327.1996.tb00719.x

[B128] Thompson L. F. Surface E. A. (2009). Promoting favorable attitudes toward personnel surveys: the role of follow-up. *Mil. Psychol.* 21 139–161. 10.1080/08995600902768693

[B129] Timmreck C. W. Bracken D. W. (1997). Multisource feedback: a study of its use in decision making. *Employ. Relat. Today* 24 21–27. 10.1002/ert.3910240104

[B130] Tomlinson G. (2010). Building a culture of high employee engagement. *Strateg. HR Rev.* 9 25–31. 10.1108/14754391011040046

[B131] Truxillo D. M. Cadiz D. M. Hammer L. B. (2015). Supporting the aging workforce: a review and recommendations for workplace intervention research. *Annu. Rev. Organ. Psychol. Organ. Behav.* 2 351–381. 10.1146/annurev-orgpsych-032414-111435

[B132] van Dierendonck D. Haynes C. Borrill C. Stride C. (2007). Effects of upward feedback on leadership behaviour toward subordinates. *J. Manag. Dev.* 26 228–238. 10.1108/02621710710732137

[B133] Vukotich G. (2014). 360^°^ feedback: ready, fire, aim - issues with improper implementation. *Perform. Improv.* 53 30–35. 10.1002/pfi.21390

[B134] Wang M. Beal D. J. Chan D. Newman D. A. Vancouver J. B. Vandenberg R. J. (2017). Longitudinal research: a panel discussion on conceptual issues, research design, and statistical techniques. *Work Aging Retire.* 3 1–24. 10.1093/workar/waw033 32257296

[B135] Ward P. (2008). Reinventing the employee survey at Fujitsu Services. *Strateg. Commun. Manag.* 12 32–35.

[B136] Welbourne T. M. (2016). The potential of pulse surveys: transforming surveys into leadership tools. *Employ. Relat. Today* 43 33–39. 10.1002/ert.21548

[B137] Wiley J. (2012). Achieving change through a best practice employee survey. *Strateg. HR Rev.* 11 265–271. 10.1108/14754391211248675

