# Supplementary material for: Following Up on Employee Surveys: A Conceptual Framework and Systematic Review
Source: Front Psychol. 2021 Dec 9;12:801073. doi: 10.3389/fpsyg.2021.801073 (PMC8696015; doi:10.3389/fpsyg.2021.801073)
Supplement: Supplementary file 1 [file Table_1.pdf]

Appendix

No.	Authors	Year	Field	Terms	Study Type/ Analytic Methods	Summary Findings	Notes
1	Adams & Sherwood	1979	Industrial and organizational psychology	Survey feedback (program); feedback meetings	Pre/post design; randomized controlled trial with control and placebo group	Mixed findings - some improvements in work efficiency, supervisory consideration, and work satisfaction, but also some declines on work satisfaction and supervisory consideration	
2	Alderfer & Ferriss	1972	Industrial and organizational psychology	Survey feedback; organizational diagnosis	Questionnaires and interviews to examine how managers perceived the feedback meetings	Higher level managers had trouble acknowledging their issues. Authors suggest that traditional work family model for feedback discussions might not be most beneficial, but instead peer group meetings first	Book chapter
3	Alderfer & Holbrook	1973	Education	Survey feedback; feedback sessions	Pre/Post design implemented in entire organization; behavior observations in feedback meetings	Different meeting structure was tested (instead of traditional work family model). Mixed results - some improvements among management in e.g., readiness for change	
4	Amba-Rao	1989	Industrial and organizational psychology	Survey feedback; employee survey; organizational diagnosis; action planning	Pre/Post design implemented in entire organization with three cycles	Overall positive results after third cycle; some improvements in communication and employee relations	

(continued)

No.	Authors	Year	Field	Terms	Study Type/ Analytic Methods	Summary Findings	Notes
5	Anderzén & Arnetz	2005	Medicine	Survey-based workplace intervention program; unit-specific and fact-based improvement work	Pre/Post design implemented in 22 work units	Overall positive results - improvements in employee wellbeing, work-related exhaustion, performance feedback, participatory management, skills development, efficiency, and leadership; a decrease on biologic stress markers and absenteeism	
6	Baker, King, MacDonald, & Horbar	2003	Medicine (Pediatrics)	Survey feedback	Pre/Post design implemented in all work units participating in quality improvement program	Great variation in intervention implementation; mixed results - some units improved, some did not	
7	Björklund, Grahn, Jensen, & Bergström	2007	Industrial and organizational psychology	Survey feedback (SF); action plans	Pre/post design implemented in four organizations; ex post facto study (groups defined by asking participants retrospectively about survey use)	Mixed results - improvements on some factors for feedback and action planning groups (e.g., leadership, commitment to organization); no changes in sick leave, job demands, and control at work	
8	Born & Mathieu	1996	Industrial and organizational psychology	Organizational development survey; feedback; action-planning	Pre/post design implemented in entire organization	Generally, using survey feedback was effective for improvement, but supervisors with low ratings tended not to use the feedback	

(continued)

No.	Authors	Year	Field	Terms	Study Type/ Analytic Methods	Summary Findings	Notes
9	Bowers	1973	Industrial and organizational psychology	Survey feedback	Pre/post design implemented in several organizations with different treatment and control groups	Survey feedback was most associated with positive changes in organizational climate in comparison to other treatments, e.g., training or consultation	
10	Burke, Coruzzi, & Church	1996	Industrial and organizational psychology	Action planning; organizational survey	Pre/post design implemented in entire organization	Only few improvements, but by second survey, predictors of employee motivation and organizational performance had aligned, which the authors interpreted as a change to a more integrated and connected organization	Book chapter
11	Callahan & Lake	1973	Education	Survey feedback	Interviews with organizational actors following survey feedback intervention	Many positive changes regarding the overall functioning of the college became visible	
12	Church, Golay, Rotolo, Tuller, Shull, & Desrosiers	2012	Industrial and organizational psychology	Employee survey; organizational survey; survey feedback; survey action planning	Pre/post design implemented in four organizations; ex post facto study (groups defined by asking participants retrospectively about survey use)	Improvements on general satisfaction; lower turnover intentions in groups that reported that their survey results were acted on	Book chapter; based on same data as Church & Oliver (2006); follow-up study

(continued)

No.	Authors	Year	Field	Terms	Study Type/ Analytic Methods	Summary Findings	Notes
13	Church, Margiloff, & Coruzzi	1995	Industrial and organizational psychology	Organizational survey; data-based feedback	Cross-sectional survey	The authors provide a prescriptive framework for identifying target areas for change within the scope of an employee survey	
14	Church & Oliver	2006	Industrial and organizational psychology	Survey feedback; survey action planning	Cross-sectional examination and for some groups pre/post design; ex post facto study (groups defined by asking participants retrospectively about survey use)	Improvements on general satisfaction, lower incidents rates of accidents, and lower turnover (intentions) in groups that reported that their survey results were acted on	Book chapter; based on same data as Church et al., (2012)
15	Conlon & Short	1984	Industrial and organizational psychology	Survey feedback; action planning	Quasi-experimental field test with pre/post design and control group	Mixed results - some changes in supervisor ratings, task perceptions, goal clarity, and opportunity for advancement; managers with higher ratings were more likely to give feedback	
16	Cooke & Coughlan	1979	Industrial and organizational psychology	Survey feedback-problem solving-collective decision (SF-PS-CD) intervention	Pre/post design; randomized controlled trial with 24 schools randomized into four groups	Overall organizational health and work attitudes of teachers improved in the intervention groups	
17	Cucina, Walmsley, Gast, Martin, & Curtin	2017	Industrial and organizational psychology	Survey key driver analysis; survey; action planning	Variety of different analyses on an archival dataset	According to authors, survey key driver analysis has many flaws and should not be used in employee survey practice	For citations of commentaries following this article, please refer to main document

(continued)

No.	Authors	Year	Field	Terms	Study Type/ Analytic Methods	Summary Findings	Notes
18	Dodd & Pesci	1977	Business	Opinion survey (feedback meeting); bottoms-up action programs	Quasi-experimental design with pre/post comparison and control group	Managers who had been trained in conducting survey feedback meetings were rated more favorably on a variety of outcomes, e.g., quality of action planning development skills	
19	Eklöf & Hagberg	2006	Ergonomics	Feedback intervention	Pre/post design; randomized controlled trial with three experimental groups and control group	Positive effects for social support when the supervisor received survey feedback by trained ergonomists. No effects were found for the other feedback groups or for other outcomes, e.g., physiological outcomes	Based on same data as Eklöf, Hagberg, Toomingas, & Tornqvist (2004)
20	Eklöf, Hagberg, Toomingas, & Tornqvist	2004	Occupational Health	Feedback intervention	Pre/post design; randomized controlled trial with three experimental groups and control group	All feedback groups showed decreases on the reported average number of workplace modifications, whereas the feedback groups decreased less than the control group	Based on same data as Eklöf & Hagberg (2006)
21	Elo, Leppänen, & Sillanpää	1998	Occupational Medicine	Survey feedback	Pre/post design implemented in selected departments	Mixed findings - one of the departments reported greater work variability and reduced mental and physical strenuousness	
22	Fraser, Leach, & Webb	2009	Industrial and organizational psychology	Employee survey (action); action plans	Semi-structured interviews with 18 managers	Identification of important factors for action, e.g., clear action purpose, senior management endorsement, and support of trained change agents	

(continued)

No.	Authors	Year	Field	Terms	Study Type/ Analytic Methods	Summary Findings	Notes
23	Fridner, Pingel, Løvseth, Sendén, & Schenck-Gustafsson	2014	Healthcare	Survey feedback method; action	Case study description	Description of a survey feedback implementation in a hospital with physicians participating in the feedback meetings	
24	Gable, Chyung, Marker, & Winiecki	2010	Industrial and organizational psychology	Employee engagement survey; actions	Unstructured interviews with some participants, followed by cross-sectional survey of larger sample	Leaders who received survey results from their superiors through multiple communication channels found the survey most useful	
25	Gavin	1984	Industrial and organizational psychology	Survey feedback program (SFP)	Case study description	Mixed findings - some improvements, but study was heavily confounded by early termination due to company refusing collaboration	
26	Gavin & Krois	1983	Industrial and organizational psychology	Survey feedback program (SFP)	Feedback implementation with observations from facilitators	Different types of groups (age, tenure, education) showed different patterns of topics discussed and time spent discussing them during feedback sessions	
27	Gavin & McPhail	1978	Industrial and organizational psychology	Data feedback; diagnostic questionnaire	Quasi-experimental design with pre/post comparison and without control group	Most improvements were achieved on measures of specific topics regarding the work setting rather than general organizational climate measures	
28	Griffin, Hart, & Wilson-Evered	2000	Industrial and organizational psychology	Employee opinion surveys	Pre-post design implemented in entire organization	Improvements followed the feedback intervention on most measures, e.g., leadership, workplace morale, recognition, and appraisal	Book chapter

(continued)

No.	Authors	Year	Field	Terms	Study Type/ Analytic Methods	Summary Findings	Notes
29	Hautaluoma & Gavin	1975	Industrial and organizational psychology	Organizational diagnosis; feedback interventions	Pre/Post design implemented in entire organization	Improvements were found for attitudes, turnover, and absenteeism, but with heavy reliance on consultants to conduct the intervention	
30	Huebner & Zacher	2021	Industrial and organizational psychology	Employee survey; action planning	Pre/post design implemented in entire organization; ex post facto study (groups defined by whether they entered action plans into online system)	Improvements were found for employee attitudes when action plans were developed, but effects were generally small	
31	Jöns	2000	Industrial and organizational psychology	Self guided feedback processes (FBP)	Pre/Post design implemented in three organizations with three different groups	Leadership ratings and previous feedback experiences were identified as moderators for survey feedback meetings	Book chapter
32	Jury, Goh, Olsen, Elston, & Phillips	2009	Healthcare	Action-oriented staff survey; staff opinion survey; action plans	Pre/Post design implemented in healthcare system	Intervention led to a variety of improvements regarding workplace culture	Based on same data as Jury, Machin, Phillips, Goh, Olsen, & Patrick (2009)
33	Jury, Machin, Phillips, Goh, Olsen, & Patrick	2009	Healthcare	Action-oriented staff survey; staff opinion survey; action plans	Pre/Post design implemented in healthcare system	Description of action planning process (including issues); for results see Jury, Goh, Olsen, Elston, & Phillips (2009)	Based on same data as Jury, Goh, Olsen, Elston, & Phillips (2009)

(continued)

No.	Authors	Year	Field	Terms	Study Type/ Analytic Methods	Summary Findings	Notes
34	Kennedy	1994	Industrial and organizational psychology	Employee survey; action planning	Pre/post design implemented in entire organization	Departments that implemented changes based on the survey results showed improvements on turnover, safety, absenteeism, and a variety of other measures, e.g., supervision, communication	
35	Klein, Kraut, & Wolfson	1971	Industrial and organizational psychology	Employee attitude survey feedback	Natural experiment; four study groups	Variables such as quality of meetings, person presenting the information, and number of meetings influenced how satisfied participants were with the feedback process and data utilization	
36	La Grange & Geldenhuys	2008	Economics & Management	Feedback intervention	Pre/Post design implemented in selected departments	Some departments showed improvements on some organizational culture facets, but generally effects were small	
37	Mann	1957	Industrial and organizational psychology	Survey feedback; attitude and opinion survey	Pre/Post design implemented in selected departments; two feedback departments and two control departments	The greater the involvement of all organizational members in the feedback sessions, the greater the achieved change in employee attitudes	Book chapter; based on same data as Mann (1961)*
38	Mann	1961	Industrial and organizational psychology	Survey feedback; attitude and opinion survey	Pre/Post design implemented in selected departments; two feedback departments and two control departments	The greater the involvement of all organizational members in the feedback sessions, the greater the achieved change in employee attitudes	Book chapter; based on same data as Mann (1957)*

Note: *Overlap of data is assumed, but could not be confirmed with author(s)

(continued)

No.	Authors	Year	Field	Terms	Study Type/ Analytic Methods	Summary Findings	Notes
39	Mann & Likert	1952	Applied Social Sciences / Anthropology	Company-wide study of employee and supervisor attitudes and opinions	Case study description	Important factors for survey-based change were high involvement, group discussions, top management commitment, and personal analysis of survey data	
40	McElvaney & Miles	1971	Education	Survey feedback	Pre/Post design implemented in schools	Mixed findings - some improvements in e.g., communication, but other measures did not change; overall small changes	Book chapter; based on same data as Miles et al. (1969)*
41	Miles, Hornstein, Callahan, Calder, & Schiavo	1969	Industrial and organizational psychology	Feedback; action planning	Pre/Post design implemented in schools	Some short-term changes were achieved (e.g., in communication and interpersonal relationships), but did not last due to a lack of follow-up	Book chapter; based on same data as McElvaney & Miles (1971)*
42	Nadler, Cammann, & Mirvis	1980	Industrial and organizational psychology	Survey feedback	Quasi-experimental design with pre/post design and control groups	Only some work groups improved on measures such as e.g., satisfaction with work, but in branches that used the feedback system poorly, negative effects arose	Based on same data as Nadler, Mirvis, & Cammann (1976)
43	Nadler, Mirvis, & Cammann	1976	Industrial and organizational psychology	Survey feedback	Quasi-experimental design with pre/post comparison and control group	Only some work groups improved on measures such as e.g., satisfaction with work, but in branches that used the feedback system poorly, negative effects arose. Also, differences between different work groups emerged	Based on same data as Nadler, Cammann, & Mirvis (1980)

Note: *Overlap of data is assumed, but could not be confirmed with author(s)

(continued)

No.	Authors	Year	Field	Terms	Study Type/ Analytic Methods	Summary Findings	Notes
44	Neff	1966	Sociology	Survey feedback; actions	Case study	Description of implementation of survey feedback in an organization, with focus on the role of the social scientist supporting the intervention	Book chapter
45	Ogilvie	1978	Education	Survey feedback intervention	Case Study	Many different change interventions were implemented to improve communication and collaboration in school among school staff, but also teachers and students	
46	Peter	1994	Healthcare (Nursing)	Survey feedback; action plans	Pre/Post design implemented in selected departments	Post measures could not be obtained due to administrative changes to the organization, but nursing managers reported high satisfaction with the intervention and the wish to use it again	Based on same data as Peter, Lytle, & Swearengen (1997)*
47	Peter, Lytle, & Swearengen	1997	Healthcare (Nursing)	Survey feedback; action plans	Pre/Post design implemented in selected departments	Mixed findings - some units showed some improvements; job satisfaction increased significantly in one work unit	Based on same data as Peter (1994); follow-up study*
48	Rafferty & Griffin	2001	Industrial and organizational psychology	Organizational diagnosis; employee opinion survey	Pre/Post design implemented in entire organization	Mixed findings - higher intensity of change activities regarding leadership led to improvements in leadership perceptions, whereas this was not the case for perceptions of feedback and workplace morale	

Note: *Overlap of data is assumed, but could not be confirmed with author(s)

(continued)

No.	Authors	Year	Field	Terms	Study Type/ Analytic Methods	Summary Findings	Notes
49	Solomon	1976	Industrial and organizational psychology	Survey feedback O.D. technique	Pre/Post design implemented in selected departments	Teams with a lot of conflict, in which a participative management style was seldom used, or with low ratings on fairness, benefitted most from the survey feedback intervention	
50	Swanson & Zuber	1996	Human Performance	Employee survey	Case study	Employee survey intervention failed due to the hostile organizational culture and an unwillingness of management to engage in the process or use the data	
51	Ward	2008	Communication	Employee survey; opinion survey; follow-up action planning	Case study	An employee survey was successfully implemented at Fujitsu as the change initiative fit the organizational culture well; employee engagement improved	
52	Werner	2004	Industrial and organizational psychology	Employee survey; staff survey	Case study	Company implemented top-down and bottom-up measures in response to employee survey and saw improvements	Book chapter
53	Wiley	2012	Industrial and organizational psychology	Employee survey; action planning	Cross-sectional survey of 31 survey practitioners	Barriers to effective action planning include a lack of proper execution, lacking support from top management, and a lack of resources	
